# A WeChat-based smoking cessation intervention for Chinese smokers: A pilot study

**DOI:** 10.1016/j.invent.2022.100511

**Published:** 2022-02-23

**Authors:** Ting Luo, Mirandy S. Li, Donna Williams, Jackson Fritz, Kaylin Beiter, Stephen Phillippi, Qingzhao Yu, Stephen Kantrow, Wei-Ting Lin, Yu-Hsiang Kao, Yongchun Chen, Liwei Chen, Tung-Sung Tseng

**Affiliations:** a Moores Cancer Center, University of California San Diego, La Jolla, California 92122, USA; b Behavioral and Community Health Sciences, School of Public Health, Louisiana State University Health Sciences Center-New Orleans, New Orleans, LA 70122, USA; c School of Medicine, Louisiana State University Health Sciences Center-New Orleans, New Orleans, LA 70122, USA; d Biostatistics, School of Public Health, Louisiana State University Health Sciences Center-New Orleans, New Orleans, LA 70122, USA; e Department of Global Community Health and Behavioral Sciences, School of Public Health & Tropical Medicine, Tulane University, New Orleans, LA 70122, USA; f Department of Clinical Nutrition, Henan Provincial People's Hospital, Zhengzhou University People's Hospital, Zhengzhou, Henan 450003, China; g Department of Epidemiology, Fielding School of Public Health, University of California Los Angeles, Los Angeles, California 900095, USA

**Keywords:** Tobacco, Smoking cessation, WeChat, China, Smokers, Social media

## Abstract

**Background:**

China is the largest tobacco producer and has the highest number of tobacco consumers in the world. Extensive research has demonstrated the utility of social media for smoking cessation. WeChat is the most commonly used social media platform in China, but has not yet been utilized for smoking cessation interventions. The objectives of this study are (1) to evaluate the efficacy of a WeChat-based smoking cessation intervention; and (2) to examine a possible additive effect of integrating oral health and smoking-related information into a tailored, Transtheoretical Model (TTM) guided smoking cessation intervention.

**Methods:**

Eligible adults were recruited through WeChat from July 1 to August 6, 2019, to participate in a 3-arm, single-blinded, randomized controlled trial. We enrolled and randomized 403 participants into three groups: the Standard Group, Enhanced Group, or a Waitlist-Control Group. Participants in the Standard Group received 20 smoking cessation-related messages for 2 weeks; participants in the Enhanced Group received this same protocol plus 6 oral health-related messages over an additional week. Participants in the Control Group received smoking cessation-related messages, after the post-intervention assessment. The primary outcome was TTM Stage of Change, and the secondary outcomes were 7-day Point Prevalence Abstinence (PPA), 24-h PPA, daily cigarette use, and nicotine dependence at 4 weeks follow-up post intervention, comparing intervention groups with the control group. The overall program attrition rate was 46%. Paired *t*-tests, McNemar tests, and linear and logistic regression were used to examine differences in smoking cessation outcomes within and between groups.

**Results:**

Participants in the Enhanced Group (β = −1.28, 95%CI: −2.13, −0.44) and the Standard Group (β = −1.13, 95%CI: −1.95, −0.30) reported larger changes in nicotine dependence scores, compared to participants in the Waitlist Group. No statistically significant differences were found between the Enhanced Group and the Standard Group.

**Discussion:**

This WeChat-based intervention was effective for smoking cessation overall. The addition of oral health information did not significantly improve the intervention.

## 1 Introduction

Tobacco use is associated with many cancers and other detrimental health conditions, and complications from these conditions results in millions of lives and hundreds of billions of dollars lost each year worldwide (CDC, 2010). Though smoking prevalence has significantly declined worldwide over the past several decades, especially in developed countries such as the United States, prevalence remains high in some developing counties, such as China (WHO, 2017). According to the Global Adult Tobacco Survey (GATS 2018), 26.6% of people in China ages 15 and above were smokers, including 50.5% (300 million) of males and 2.1% of females (WHO, 2018). The China City Adult Tobacco Survey 2013–14 showed that smoking prevalence among individuals living in 14 cities ranged from 17.7% to 24.5% (WHO, 2015). In addition, cessation efforts among current smokers remain low: less than one in five current smokers thought about quitting (16.1%) or made a quit attempt (19.8%) in the past 12 months (WHO, 2018). Only 15.6% of ever daily smokers had quit (WHO, 2018). The quit ratio, defined as the percentage of former daily tobacco smokers among ever daily tobacco smokers, is 12.8%, which is the second-lowest among all WHO GATS countries (Yang et al., 2011a). Furthermore, less than 10% of smokers who attempted to quit used assistive measures (WHO, 2018); in comparison, in the US, 57.2% of smokers were advised by a health professional to quit and 31.2% used cessation counseling and/or medication when trying to quit (CDC, 2017). Moreover, of Chinese smokers who have tried to quit, 33.2% relapsed (Yang et al., 2011a). All of these findings suggest that Chinese smokers may have a lower interest in quitting and those that do attempt to quit are unable to find or use cessation assistance.

China also has some of the highest prevalence of both smoking and oral disease in the world (Yang et al., 2011a; Yang et al., 2011b). The high prevalence of oral disease may be partly attributed to low oral health awareness, but is compounded by the fact that tobacco use is a major cause of oral disease burden (Dy et al., 2011). Among all factors that contribute to oral diseases, smoking cannot be underestimated (Dy et al., 2011). Smoking impacts every part of body, thus an increase in general health awareness may increase the intent to quit smoking (Dy et al., 2011). Furthermore, increasing oral health awareness can increase the chance of receiving teeth cleanings and dental checks, which may further increase general health awareness (Dy et al., 2011). Thus, the additional inclusion of oral health information has the potential ability to increase smoking cessation rates and possibly decrease oral disease prevalence at the same time. Moreover, quitting smoking can lower the risk of periodontitis and other oral diseases, which has led dental health providers to promote smoking cessation (Fiorini et al., 2014). However, limited studies have examined the effect of promoting oral health awareness on improving smoking cessation outcomes (Dawson et al., Dec 2013).

Many effective strategies have been developed in the US and in China to help individuals quit smoking, including increased taxes (Bader et al., 2011), restrictions on smoking in public spaces (Fichtenberg and Glantz, 2002), warning labels (Hammond et al., 2004), health professional advice (GeneralSurgeon, 2014), counseling (TCPG, 2008), quitlines (Schauer et al., 2013), medications (ALS, 2018), and anti-tobacco mass media campaigns (WHO, 2013). Recently, social media has emerged as an important avenue for novel smoking cessation interventions (Luo et al., 2021; Naslund et al., Oct 2017). Social media is easily accessible, regardless of where smokers live, which affords the opportunity to engage with large and diverse populations. Social media also allows for increasing interactions between smokers and interventionists, due to the limited impact of logistical barriers such as travel. Social media interventions may further have the ability to provide tailored information and promote peer/social/emotional support. Facebook and Twitter have been demonstrated to be effective intervention tools for smoking cessation (Luo et al., 2021; Naslund et al., Oct 2017). For political reasons however, people in China cannot access these social media platforms. An alternative widespread social media platform that is available in China is WeChat, which combines the major functions of Facebook, WhatsApp, Twitter, and PayPal (Brennan, 2017; Tencent, 2017). WeChat was developed by the Tencent Company in China, was first released on January 21, 2011, and has become one of the most utilized social media platforms in China. In 2020, WeChat had over 1.2 billion monthly active users, including 1.4 million monthly active users from the US (Content, 2021). WeChat is widely used in work environments, where 70% of Chinese depend on WeChat instead of e-mail for work-related communication (Content, 2021). However, the application of WeChat for use in smoking cessation interventions is limited. In addition, previous literature has indicated that theory-based smoking cessation interventions may result in better smoking cessation outcomes (Glanz, 2015). The Transtheoretical Model (TTM) has been demonstrated to be effective in guiding smoking cessation interventions (Glanz, 2015). Therefore, the objectives of this study are to evaluate the efficacy of a pilot WeChat-based smoking cessation intervention and to examine an additive effect of integrating oral health and smoking-related information into a tailored, TTM-guided smoking cessation intervention.

## 2 Methods

### 2.1 Participants and recruitment

A new WeChat account (ID: QuitSmokingHelp) was created for smoking cessation services and participants were able to “friend” the account online. On July 1st, 2019, recruitment advertisements were posted on the WeChat Official Account (through the Chinese Clinical Nutrition Network). Advertisement information can be found in Supplementary 1. Smokers could “friend” the interventionist via their QR code, WeChat ID, or through a phone call or email. After prospective participants contacted and “friended” the interventionist on WeChat, their eligibility was assessed. Inclusion criteria included a) current smokers; b) living in China; c) 18 years and older; d) active users of WeChat (login at least once a day); and e) indicated willingness to participate in the study (written consent). Exclusion criteria included a) non-smokers or former smokers; b) under 18 years of age; c) unable to read and type in Chinese using a smartphone or computer; d) not living in China; e) not active users of WeChat; or f) not willing to consent to participate in the study. Consenting individuals were asked for the last 4 digits of their most frequently used cell phone number, which was used as their “Smoker ID.” The Smoker ID was used to link pre-intervention, process evaluation, and post-intervention surveys. Participants who completed either the baseline assessment, process evaluation, immediately after intervention assessment, and/or post-intervention assessment received financial incentives through a corresponding “Red Packet” (a direct payment feature of WeChat).

**Fig. 1:**
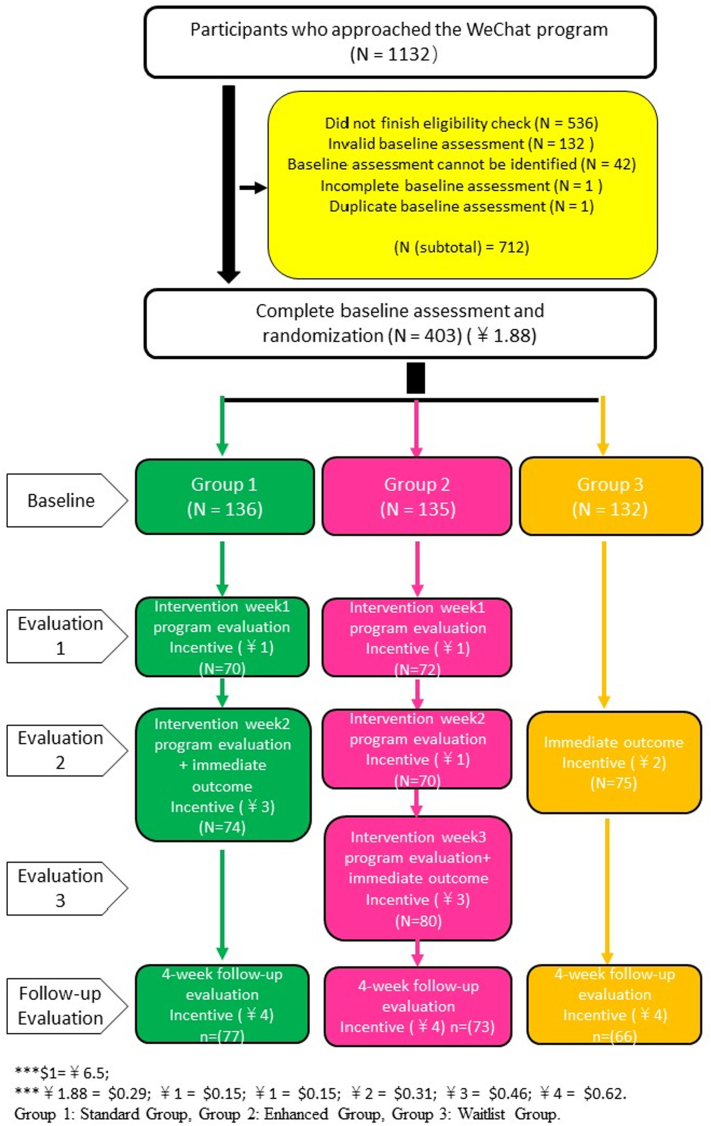
Flow chart of randomization.

Between July 1 to August 5, 2019, of the 1132 people who “friended” our project WeChat-“QuitSmokingHelp”, 729 were excluded due to ineligibility, submitted an incomplete or invalidated baseline survey, or did not provide written consent. Thus, 403 people were eligible and consented to participate in the study. A total of 136, 135, and 132 smokers were randomly assigned to the Standard Group, Enhanced Group, and Waitlist Group, respectively. After the intervention, 77, 73, and 66 participants remained in the Standard Group, Enhanced Group, and Waitlist Group, respectively. Further details on the selection, assignment, and incentive process are shown in Fig. 1.

### 2.2 Study design

A non-probability convenience sampling method was used for this study. After completing the online baseline assessment, all consenting, eligible smokers were randomly distributed to participate in a 3-arm, single-blinded, randomized controlled trial (RCT). Participants in intervention group 1 (Standard Group) received 20 smoking-related messages for 2 weeks; participants in intervention group 2 (Enhanced Group) received 20 smoking-related messages for 2 weeks and an extra 6 oral health-related messages for an additional week. Participants in the group 3 (Waitlist Group) received smoking-related messages after the post-intervention assessment.

### 2.3 Intervention content categories and processes

The conceptual model for this study (Supplementary 2) is based on the TTM, which has been used extensively in a variety of health behavior change studies including smoking cessation (Glanz, 2015). Six intervention content categories were established in keeping with TTM constructs: consciousness-raising, self-efficacy, helping relationships, coping skills, stimulus control, and oral health awareness (Glanz, 2015; Prochaska and DiClemente, Jun 1983). In addition, we adapted some materials from the smoking counseling material of the Louisiana Tobacco Control Initiative (LATCI, 2021). Intervention messages included videos, images, and texts.

The interventionist (“QuitSmokingHelp”) delivered intervention messages every weekday at 8:00 am-9:00 am (China Standard Time, GMT +8) via WeChat (version 7.0.3) broadcasting messages. Detailed delivery information can be found in Supplementary 3. Every Friday night (in China Standard Time, GMT +8), a text-only discussion session for each group was facilitated by the interventionist through the “QuitSmokingHelp” account. The group discussion was related to the content that was delivered during that week, with a duration of around 30 min. Participants were able to ask smoking-related questions and received answers from the interventionist in a timely manner. Participants were also able ask the interventionist any smoking related questions outside of the group discussion until the 4-week follow-up assessment, with the interventionist responding within 24 h. A more detailed description of the intervention can be found in our article outlining the implementation of this pilot study (Luo et al., 2021a).

### 2.4 Measurements

Stages were defined based on the TTM model. A smoker's stage of readiness before “action” has typically been assessed during past interventions with the question “When do you intend to quit smoking?” (Prochaska et al., 1988; Biener and Abrams, 1991) Smokers who responded “do not intend to quit” in our study were categorized as being in the precontemplation stage; those who responded “within next the 6 months” were categorized as being in the contemplation stage; those who responded “within next the 30 days” were categorized as being in the preparation stage (Prochaska et al., 1988; Biener and Abrams, 1991). Smokers who responded “No” to the question, “Have you smoked any cigarettes in the past 7 days, even a puff?” were categorized as being in the action stage (Prochaska et al., 1988; Biener and Abrams, 1991). 7-day point prevalence abstinence rate was defined as the number of smokers who self-reported quitting smoking for 7 days divided by the total number of smokers. The measurement of 7-day PPA is the same as the measurement of the action stage (Hughes et al., 2003). Smokers who reported a 7-day abstinence were categorized in the action stage. Similarly, 24-h PPA was measured using the question “Have you smoked any tobacco products in the last 24 hours?” (Hughes et al., 2003) 24-h point prevalence abstinence rate was defined as the number of smokers who self-reported quitting smoking for 24 h divided by the total number of smokers. Daily cigarette use was measured based on a question from the survey of nicotine dependence, using a multiple-choice question with 4 answers: 1) 10 or less, 2) 11–20; 3) 21–30; or 4) 31 or more (Etter et al., 1999). Nicotine dependence was determined through the Fagerstrom Test for Nicotine Dependence, a previously-validated 6-item questionnaire (Etter et al., 1999). Theoretical concepts and oral health awareness constructs were measured by previously validated questionnaires (Supplementary 4).

Assessments of our intervention's effect utilized questionnaires at pre-intervention and at 4-weeks post-intervention. Survey questions collected information including participant demographics, outcome measurements, content measurements, and other various information (detailed information can be found in the measurements subsection). In addition, participants were required to complete a process evaluation survey questionnaire, which was conducted during the 3-week intervention period. All assessment surveys were delivered via “Wenjuanwang”, a popular China marketing research tool (Kuo, 2018). For each questionnaire, participants were identified via a unique Smoker ID that they were required to fill in at the start of the survey.

### 2.5 Analyses

1) Sample size estimation and power analysis

Few social-media based randomized controlled trials have adopted TTM stage progression as their outcome of interest. Previous social media-based randomized controlled trials for smokers who were motivated to quit have suggested that quit rates (24-h and 7-day quitting rates) for participants may range from 5% to 40% (Pechmann et al., 2015a; Ramo et al., 2015a; Ramo et al., 2015b; Pechmann et al., 2017; Naslund et al., 2017; Taylor et al., 2017). Thus, we estimated a 10% stage progression for the Waitlist Control Group, 20% stage progression for the Standard Group, and 30% stage progression for the Enhanced Group. The Chi-square effective size was estimated to be 0.2041. We used PASS software version 11 to calculate the minimal required sample size. To achieve a minimal power level of 0.80 with a type I error probability (significance level) of 0.05, a sample size of n = 232 was considered necessary. Based on previous social media-based smoking cessation intervention studies, we expected an attrition rate between 20% and 50% (Danaher et al., Sep 2013; Naughton et al., 2014; YTD et al., 2015; Pechmann et al., 2015b; Ramo et al., 2015c; Baskerville et al., Mar 2016; Pechmann et al., Mar 2016; Kim et al., 2017), and allowed for a 30% attrition rate in our sample size calculations. Overall, we projected a minimal required sample size of n = 330.

2) Statistical analyses

Descriptive statistics were used to compare the baseline participants' characteristics between the 3 intervention groups. Chi-square/Fishers exact tests were used for comparison among categorical variables and ANOVA for continuous variables. Two tailed tests were performed with the significance level at 0.05. Continuous outcomes included stage of change at follow-up and the change in nicotine dependence score. Questionnaire responses were scored with stages of change being sequential: smokers in the pre-contemplation stage were scored as 1, smokers in the contemplation stage were scored as 2, smokers in the preparation stage were scored as 3, and smokers in the action stage were scored as 4. Change in Nicotine Dependence score (Fagerstrom test) was obtained from the difference between follow-up and baseline assessments. The maximum range was between −10 to 10, though in this study we only observed a range of change between −8 to 7.

Linear regressions were conducted to compare these continuous outcomes among groups adjusting or not adjusting for other covariates. Logistic regressions were applied to compare dichotomous outcomes among groups, including change in 7-day PPA, change in 24-h PPA, and change in daily cigarette use. Change in 7-day PPA was classified into two categories: progressed and did not progress. “Progressed” referred to those who changed from “has smoked in the past 7 days” at baseline to “has not smoked in the past 7 days” at follow-up. Did not progress included “regressed”, “no change (smoking)”, and “no change (non-smoking)”. “Regressed” referred to those who changed from “has not smoked in the past 7 days” at baseline to “has smoked in the past 7 days” at follow-up; “no change (smoking)” referred to no change in “has smoked in the past 7 days” at baseline and follow-up; and “no change (non-smoking)” referred to no change in “has not smoked in the past 7 days” at baseline and follow-up. Change in 24-h PPA used the same definition as change in 7-day PPA but over the past 24 h. Change in daily cigarette use was classified into “reduced” and “did not reduce”. “Reduced” referred to those who did not smoke in the past 7 days or who moved at least one stage towards lighter cigarette use; “did not reduce” referred to those who moved at least one stage towards heavier cigarette use or who reported no change in daily cigarette use.

Intervention effects were examined using regression by comparing intervention groups (the Enhanced Group and the Standard Group) and the Waitlist group; addictive effect (additional oral health awareness content effect) was examined using regression by comparing two intervention groups: the Enhanced Group versus the Standard Group. A significant coefficient for the indicator variables would have indicated a significant difference between the different groups. We analyzed cessation outcomes in crude models and adjusted models. For the adjusted models, all demographic variables (age, gender, education level, income level, and self-reported living area) were included into the final regression model as covariates. According to previous literature, weight gain is common among smokers who quit smoking, and BMI has been demonstrated to influence smoking cessation outcomes (Ussher et al., 2014); thus, this study also included BMI into the adjusted regression model as a covariate. Age at smoking initiation has also been shown to affect an individual's smoking cessation outcomes; therefore, age at smoking initiation was also included as a covariate in the final model. To avoid missing potential confounders, follow-up scores for theoretical concepts and oral health construct that were significant at the alpha = 0.05 level were included in the final model.

3) Attrition

Supplementary 5 shows demographic information and smoking behaviors comparisons according to participant completion/attrition status. The only significant difference out of 14 indicators was in sex (male: 92.6% for follow-up vs. 84.5% for attrition). Participants who completed the follow-up assessment showed similar demographic characteristics and smoking behaviors as those who did not complete the follow-up assessment. Thus, the missing data were considered as missing at random, and we concluded that the study results were not influenced much by attrition. Our primary analysis strategy is based on completed case analysis, but we also included an intent-to-treat analysis for comparison.

### 2.6 Ethical approval and consent to participate

This study was not pre-registered with any clinical trial database. However, though we did not apply for clinical trial registration, our study procedures did follow all standards for ethical guidelines of research as set out by the Louisiana State University Health Sciences Center-New Orleans (LSUHSC) Institutional Review Board (IRB#: 19–901), including the Declaration of Helsinki, the Belmont Principles, and the guidelines for ethical clinical trial research. All smokers consented to participate to study in writing by checking the YES box prior to proceeding to the baseline assessment.

## 3 Results

### 3.1 Study participants

Table 1 shows demographic information for each group at baseline. The mean and standard deviation (SD) for participants' overall age was 30.5 (9.6) and the mean (SD) of age at smoking initiation for all participants was 18.1 (4.1). The majority of participants (93.9%, n = 260) were less than 40 years old. 88.8% (n = 358) of the participants were male. 36.0% (n = 145) of participants had an annual household income under ￥50,000 ($7200) and 29.1% (n = 122) of participants earned between ￥50,000–99,999 ($7200–15,000). Most participants (57.8%, n = 233) reported living in an urban area. Approximately four in ten participants had a high school or lower education. About six in ten participants were married. 44.9% (n = 181) of participants worked in business. Approximately six in ten participants were underweight or normal weight.

**Table 1:** Demographic information at baseline.

Variables		Total (N = 403)		Standard Group 1 (N = 136)		Enhanced Group 2 (N = 135)		Waitlist Group 3 (N = 132)		P-value
		N	%	N	%	N	%	N	%	
Age (mean, SD)		30.5	9.6	29.9	9.0	31.4	9.6	30.2	10.2	0.42
Age category										0.64
	18–24	121	30.8	42	32.1	34	25.6	45	35.2	
	25–29	100	25.5	37	28.2	35	26.3	28	21.9	
	30–39	108	27.6	33	25.2	41	30.8	34	26.6	
	≥40	63	16.1	19	14.5	23	17.3	21	16.4	
Sex										0.23
	Male	358	88.8	117	86.0	119	88.2	122	92.4	
	Female	45	11.2	19	14.0	16	11.8	10	7.6	
Annual household income^a^ in ￥										0.30
	≤49,999	145	36.0	58	42.7	40	29.6	47	35.6	
	50,000-99,999	122	29.1	31	22.8	43	31.9	42	31.8	
	100,000-199,999	96	22.9	31	22.8	37	27.4	27	20.5	
	≥200,000	50	11.9	16	11.8	15	11.1	16	12.1	
Self-reported living area^b^										0.93
	Urban	233	57.8	79	58.1	80	59.3	74	56.1	
	Suburban	107	26.6	38	27.9	33	24.4	36	27.3	
	Rural	63	15.6	19	14.0	22	16.3	22	16.7	
Education level										0.87
	High school or less	153	38.0	51	37.5	55	40.7	47	35.6	
	Associated college	129	32.0	44	32.4	39	29.0	46	34.9	
	College and above	121	30.0	41	30.2	41	30.4	39	29.6	
Marital status										0.73
	Married	242	60.0	78	57.4	83	61.5	81	61.4	
	Single^c^	161	40.0	58	42.7	52	38.5	51	38.6	
Occupation										0.23
	Business^d^	181	44.9	70	51.5	62	45.9	49	37.1	
	Government/agency officers/professional staff^e^	75	18.6	24	17.7	22	16.3	29	22.0	
	Labor workers^f^	54	13.4	19	14.0	16	11.9	19	14.4	
	Self-employed and other^g^	93	23.1	23	16.9	35	25.9	35	26.5	
BMI^h^										0.58
	Underweight and normal weight	231	57.6	81	60.0	73	54.1	77	58.8	
	Overweight and obese	170	42.4	54	40.0	62	45.9	54	41.2	
Age at smoking initiation (Mean, SD)		18.1	4.1	18.0	3.6	18.3	4.6	17.9	4.0	0.76

a The current exchange rate for USD to RMB is as follows: ￥6.5 = $1; ￥ < 20,000 ≤$3077; ￥ 20,000–49,999 = $3077–$7692; ￥ 50,000–99,999 = ($7692–$15,384); ￥100,000–199,999 = $15,384–$30,769; ￥ >200,000 ≥ $30,769.

b “Urban” includes people who are living in prefecture-level cities or county-level cities; “Suburban” includes people who are living in the areas beyond a city's border; “Exurban” includes people who are living in towns; and “Rural” includes people who are living in villages.

c “Single” includes never married, widowed, divorced, and living with partner.

d “Business” includes managers, general office staff, and business service workers (e.g., salesmen, shop clerks, waiters, etc).

e “Professional Staff” includes doctors, teachers, lawyers, journalists, etc.

f “Labor Workers” includes factory workers, and farmers/foresters/fishermen.

g “Self-Employed and Other” includes self-employed, freelancers, retired, unemployed, students, and others.

h Asian BMI standards are as follows: Underweight and Normal Weight (BMI ≤ 22.9), Overweight and Obese (BMI ≥ 23).

### 3.2 Smoking cessation outcomes comparison

Table 2 reports the tobacco use outcomes using both a completed case analysis and an intent-to -treat analysis. The intent-to-treat analysis considered all participants who left the intervention as smokers who did not quit, biasing the results towards the null (that the intervention was not effective). The missing data was considered as missing completely at random, thus the following analysis results is based on completed case analysis.

**Table 2:** Tobacco use outcomes assessments using completed case analysis and intention to treat analysis.

		Complete case analysis	Intention to treat analysis
	Baseline	4 weeks at follow-up	4 weeks at follow-up
Stage of change at follow-up (4 stages, range 1–4, Mean (SD))			
Standard group	2.39 (0.61)	2.65 (0.77)	2.56 (0.72)
Enhanced group	2.49 (0.60)	2.79 (0.80)	2.64 (0.73)
Waitlist group	2.39 (0.60)	2.39 (0.74)	2.42 (0.64)
7-day abstinence (n (%))			
Standard group	0	12/77 (15.6)	12/136 (8.8)
Enhanced group	0	15/73 (20.6)	15/135 (11.1)
Waitlist group	0	7/66 (10.6)	7/132 (5.3)
24-h abstinence (n (%))			
Standard group	11/136 (8.1)	27/77 (35.1)	27/136 (19.9)
Enhanced group	6/135 (4.4)	30/73 (41.1)	30/135 (22.2)
Waitlist group	4/132 (3.0)	17/66 (25.8)	17/132 (12.9)
Daily cigarette use (10 or less) (n (%))			
Standard group	53/136 (39.0)	55/77 (71.4)	55/136 (40.4)
Enhanced group	47/135 (34.8)	54/73 (74.0)	54/135 (40.0)
Waitlist group	62/132 (47.0)	37/66 (56.1)	37/132 (28.0)
Change in nicotine dependence (range: −8-7, Mean (SD))			
Standard group	5.17 (2.68)	4.38 (2.90)	3.44 (2.63)
Enhanced group	5.37 (2.43)	4.26 (2.64)	3.49 (2.42)
Waitlist group	5.18 (2.22)	4.99 (2.43)	4.84 (2.69)

**Fig. 2:**
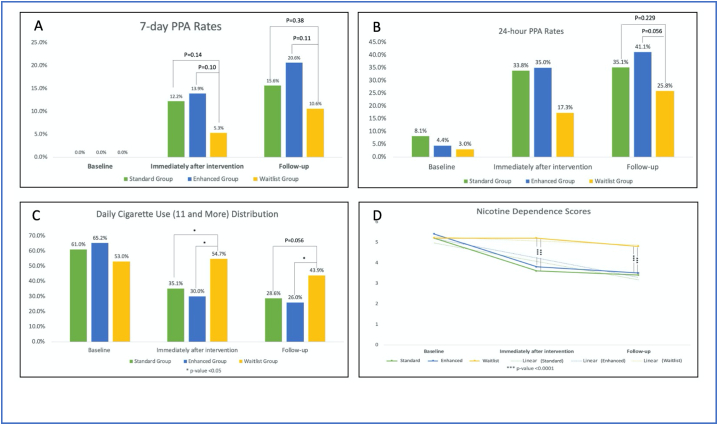
Smoking cessation outcomes at baseline, immediately after intervention, and follow-up A) 7-day PPA rates at baseline, immediately after intervention, and follow-up; B) 24-h PPA rates at baseline, immediately after intervention, and follow-u; C) Daily cigarette use distribution; D) Nicotine dependence.

Fig. 2(A) shows differences in 7-day PPA rates between groups at baseline, immediately after the intervention, and at 4-week follow-up. No statistically significant differences were found between groups across all time points. Participants from the Standard Group and Enhanced Group had higher 7-day PPA rates than the Waitlist Group (15.6%, 20.6%, vs. 10.6%, respectively) when measured at 4-week follow-up.

Fig. 2(B) shows differences in 24-h PPA rates between groups at different time points. Similar to 7-day PPA rates, no statistically significant differences were found between groups at baseline or immediately after the intervention. At 4-week follow-up, the 24-h PPA rates between the Enhanced Group and Waitlist Group were 41.1% and 25.8%, respectively (P = 0.056).

Fig. 2(C) shows the distribution of smoking 10 or more cigarettes a day, between groups, at different time points. Overall, compared to baseline, all participants immediately after the intervention and at follow-up reported lower rates of smoking 10 or more cigarettes a day. Participants from the Enhanced Group had statistically significant lower rates of smoking 10 cigarettes or more compared to the Waitlist Group (26.0% vs. 43.9%, respectively, P < 0.05).

Fig. 2(D) shows nicotine dependence scores between groups at different time points. Immediately after the intervention, statistically significant differences were found in the Standard Group and Enhanced Group, compared with the Waitlist Group (3.6, 3.8, vs. 5.2, respectively). Similarly, at follow-up, statistically significant differences were found in the Standard Group and Enhanced Group, compared with the Waitlist Group (3.4, 3.5, vs. 4.8, respectively).

### 3.3 Intervention effect

Table 3 shows the impact of smoking cessation outcomes in crude and adjusted models. In the adjusted models, the difference in mean of stage of change between Enhanced Group and Waitlist Group was significant (beta = 0.25, 95% CI: 0.004–0.49). Moreover, participants in the Enhanced Group (aOR = 3.80, 95% CI: 1.63–8.85) and Standard Group (aOR = 3.29, 95% CI: 1.45–7.45) were statistically more likely to report reductions in daily cigarette use in the adjusted model, compared to the Waitlist Group. Participants in the Enhanced Group (β = −1.28, 95%CI: −2.13, −0.44) and the Standard Group (β = −1.13, 95%CI: −1.95, −0.30) reported larger changes in nicotine dependence scores, compared to participants in the Waitlist Group.

**Table 3:** The impact of smoking cessation outcomes.

Variables		Crude model			Adjusted model		
		OR/beta	95% CI	*P*-value	OR/beta	95% CI	*P*-value
Between 3 groups							
Stage of change at follow-up (4 stages, range 1–4)^a^							
	Standard group	0.24	(−0.02, 0.50)	0.07	0.17	(−0.06, 0.41)	0.15
	Enhanced group	0.39	(0.13, 0.65)	0.004	0.25	(0.01, 0.49)	0.04
	Waitlist group	Ref	Ref		Ref	Ref	
Change in 7-day PPA rate (progressed/did not progress)^b^							
	Standard group	1.56	(0.57, 4.22)	0.38	2.14	(0.60, 7.68)	0.24
	Enhanced group	2.18	(0.83, 5.74)	0.12	2.48	(0.73, 8.43)	0.15
	Waitlist group	Ref	Ref		Ref	Ref	
Change in 24-h rate (progressed/did not progress)^b^							
	Standard group	1.45	(0.68, 3.08)	0.34	1.66	(0.68, 4.01)	0.26
	Enhanced group	2.24	(1.07, 4.71)	0.03	2.16	(0.89, 5.25)	0.09
	Waitlist group	Ref	Ref		Ref	Ref	
Change in daily cigarette use (reduced/did not reduce)^b^							
	Standard group	2.47	(1.22, 4.98)	0.01	3.29	(1.45, 7.45)	0.004
	Enhanced group	3.42	(1.68, 6.97)	<0.001	3.80	(1.63, 8.85)	0.002
	Waitlist group	Ref	Ref		Ref	Ref	
Change in nicotine dependence (range: −8-7)^a^							
	Standard group	−1.01	(−1.85, −0.17)	0.02	−1.13	(−1.95, −0.30)	0.008
	Enhanced group	−1.50	(−2.35, −0.65)	0.001	−1.28	(−2.13, −0.44)	0.003
	Waitlist group	Ref	Ref		Ref	Ref	

Between 2 intervention groups							
Stage of change at follow-up (4 stages, range 1–4)^a^							
	Standard group	Ref	Ref		Ref	Ref	
	Enhanced group	0.15	(−0.10, 0.40)	0.24	0.08	(−0.15, 0.30)	0.52
Change in 7-day PPA rate (progressed/did not progress)^b^							
	Standard group	Ref	Ref		Ref	Ref	
	Enhanced group	1.40	(0.61, 3.24)	0.43	1.16	(0.41, 3.32)	0.78
Change in 24-h rate (progressed/did not progress)^b^							
	Standard group	Ref	Ref		Ref	Ref	
	Enhanced group	1.55	(0.79, 3.04)	0.21	1.30	(0.59, 2.87)	0.51
Change in daily cigarette use (reduced/did not reduce)^b^							
	Standard group	Ref	Ref		Ref	Ref	
	Enhanced group	1.55	(0.79, 3.04)	0.21	1.30	(0.59, 2.87)	0.51
Change in nicotine dependence (range: −8-7)^a^							
	Standard group	Ref	Ref		Ref	Ref	
	Enhanced group	−0.49	(−1.30, 0.33)	0.24	−0.15	(−0.94, 0.63)	0.70

a Continuous outcome: computing linear regression models, testing if beta significant different from 0.

b Categorical outcome: computing logistic regression models, testing if odd ratio (OR) significant different from 1.

When comparing the intervention impact on smoking cessation outcomes between the Enhanced and Standard Groups, no statistically significant differences were found for any smoking cessation outcomes in crude and adjusted models. Participants in the Enhanced Group reported 30% higher 24-h PPA rate progression (aOR = 1.30; 95% CI: 0.59–2.87), 16% higher 7-day PPA rate progression (aOR = 1.16, 95% CI: 0.41–3.32), and 16% higher daily cigarette use reduction progression (aOR = 1.16, 95% CI: 0.56–2.41) in the adjusted models, compared to the Standard Group.

## 4 Discussion

### 4.1 Intervention effect

This study found that participants in a WeChat based smoking cessation intervention had better smoking cessation outcomes than the control group. Specifically, participants' successes in the intervention group relative to the waitlist-control group included progress in TTM-oriented stage of quitting, reduction in daily cigarette use, and reduction in nicotine dependence. In contrast to the study's hypothesis, addition of oral health information did not significantly improve the intervention efficacy.

This study adds to a growing body of literature surrounding the use of social media to engage individuals in health behavior change. It is the first study, to our knowledge, to use WeChat for full recruitment, intervention delivery, and assessment in order to help smokers quit. Though results are promising for the use of the intervention overall, further work is needed in order to identify the exact elements that should be included into intervention content, if any, beyond the standard group messages. Our data overall provide promising proof-of-concept results for the use of the TTM in the specific context of using social media for smoking cessation.

These promising results are still preliminary though; statistically significant differences between groups for change in 7-day PPA rate and 24-h PPA rate were not observed across all three groups. Again, this may be due in part to failure to find a difference between the standard and enhanced intervention groups, either due to the oral health content not having an impact, or to the impact of inclusion of oral health content being overshadowed by the largely impactful TTM constructs. These effects are in line with previous a systematic review for social media-based smoking cessation intervention studies (Luo et al., 2021). The review showed that the duration of previous intervention studies has ranged from 21 to 100 days, with short-term interventions less likely to report change in either 7-day PPA or 24-h PPA (Luo et al., 2021). Namkoong et al. also promoted a similar type of intervention: a 21-day Facebook-based social media anti-smoking campaign (Namkoong et al., 2018). Their study did not report either 7-day PPA or 24-h PPA, but instead it reported intermediate smoking cessation outcomes, such as intention on encourage community members to stop smoking (Namkoong et al., 2018). Kim et al. developed a 4-week Facebook based smoking cessation study and examined the 7-day PPA at baseline (0%), week 1 (13%), week 2 (19%), week 3 (19%), week 4 (19%), and week 6 (25%) (Kim et al., 2017). In general, from both the literature and our own findings, it is possible that short-term cessation interventions are more likely to result in an intermediate smoking behavior change.

### 4.2 Integrating oral health information

When integrating oral health information, no statistically significant differences in smoking cessation outcomes were found between the Enhanced (smoking + oral health information) and Standard Groups (smoking information only). One of the reasons for these insignificant findings between the Enhanced Group and the Standard Group might be due to the limited sample size of this study. Compared to other studies with 3 arms, our sample size is relatively small (Luo et al., 2021). In fact, this study shows that participants in the Enhanced Group reported 30% higher 24-h PPA rate progression than in the Standard group, though the finding was not statistically significant. China has a high prevalence of oral diseases cases (Zhou et al., 2018), and it was hypothesized in this study that a population-level desire to reduce oral disease would result in improved intervention efficacy with the promotion of oral health awareness content in the enhanced intervention group. Future studies with large sample sizes are necessary to confirm if there is a significant additive effect of adding oral health education.

### 4.3 Limitations and strengths

A non-probability convenience sampling method was used for this study. Participants who “friended” the WeChat profile indicated their interest in making changes in smoking behaviors. Presumably not all WeChat users who smoke are interested in quitting. Therefore, the results may not be fully generalizable to the entire population of WeChat-using smokers in China. However, the high number of WeChat users and the high number of smokers in China nevertheless indicate the opportunity for substantial impact. Findings of this study may also be further extended by health professionals to plan, implement, and evaluate health promotion programs for other health behavior interventions, such as marijuana use or binge drinking.

Measurements for this study were based on self-reported data, without bio-chemical validation. Self-reported data may be exaggerated, as participants may experience social-desirability bias, or be embarrassed to report that they were unsuccessful in their quitting attempts. Participants may have also suffered from recall bias by forgetting the number of cigarettes they smoked per day. According to a previous social media based smoking cessation study, biochemically validated abstinence was confirmed with approximately half of participants that self-reported abstinence at each assessment point (Ramo et al., 2015c). Self-reported studies can be influenced by the participant's emotions at the time they filled out the questionnaire. If a participant felt good at the time of the survey, their answers may be more positive; conversely, if a subject felt bad at the time of the survey, answers may be more negative.

Although the abstinence rates of using WeChat for smoking cessation might be overestimated, a RCT study design was applied in this study, which may reduce any effect on the efficacy of the study. Moreover, this is a single-blind study. Participants were unaware of the different groups and did not know which group they were placed in. However, the interventionist knew the group placements, and may have inadvertently paid more attention to those participants who were in the Standard Group and the Enhanced Group, with resultant impacts on smoking cessation outcomes. In addition, the TTM stages are non-parametric, ordinal data (no discrete differences between each stage), but this is also a common way of using the TTM for behavioral research. According to our conceptual model, Stage of Change should be the primary outcome, but the results for this outcome were not statistically significant, thus we did not address Stage of Change in detail in the introduction and discussion sections. Finally, this study also does not control for the effect of local smoking cessation policies, indicating that we are not able to exclude the impact from these potential policies.

Despite these limitations, this study remains important and is characterized by the following strengths. First, utilization of the regionally ubiquitous social media platform WeChat to recruit smokers and deliver intervention allowed us to reach a large and diverse populations, such as users who are living in rural China (Luo et al., 2021b). To our knowledge, this is a novel use of WeChat for smoking behavioral health change. In addition, in order to minimize selection bias, we decided to randomly assign the eligible participants to either the Standard Group, Enhanced Group, or the control group. Thus, the even distribution of demographic variables across all 3 groups indicates that selection bias was not a factor for internal validity.

### 4.4 Implications and future studies

This WeChat-based approach for smoking cessation has the potential to benefit a considerable number of current smokers in China, who likely otherwise do not have access to other social media platforms, via delivery over a convenient electronic platform. About 10% of WeChat users are international WeChat users (Content, 2021), indicating that this approach may further help smokers who are not currently living in China. For example, this approach can provide benefits to older Chinese immigrants who are generally not active on Facebook, WhatsApp or Twitter, but are active on WeChat. Finally, this approach can be applied to non-smoking cessation programs, such as marijuana use, binge drinking, diabetes, and obesity focused interventions.

## 5 Conclusion

This study demonstrated that WeChat is an effective tool for implementing smoking cessation interventions. To our knowledge, this was one of the first study to assess the efficacy of using WeChat for smoking cessation. We also tested if integrating oral health information has the additional potential to increase program efficacy. We found that our intervention was generally effective in reducing smoking behaviors among a sample of Chinese adults. Our TTM-based standard content did not significantly benefit from addition of oral health information, and thus no statistically significant differences were found between the Enhanced and Standard Groups. Future studies are required to explore and document whether there is any true benefit to add oral health education into a smoking cessation program, and mechanisms of improved intervention efficacy. More broadly, considering continued social distancing, WeChat should be considered as a platform for smoking cessation and other behavioral interventions.

## Funding

No public funding support.

## Human rights and informed consent

All materials and procedures for this study will undergo review and approval by the Institutional Review Board (IRB) of the Louisiana State University Health Sciences Center-New Orleans (LSUHSC) (IRB#: 19–901). All smokers consented to participate to study in written by checking the YES box indicating the willingness to participate to the study before the baseline assessment.

## Welfare of animals

No animals study.

## Consent to publish

All the authors have approved the final manuscript and consented to publish our work.

## Availability of data and materials

The material that support this manuscript are available from the corresponding authors upon reasonable request.

## Declaration of competing interest

The authors declare that they have no known competing financial interests or personal relationships that could have appeared to influence the work reported in this paper.

## Appendix A Supplementary data

Supplementary material Image 1

## References

[bb0005] ALS (2018). https://www.lung.org/stop-smoking/join-freedom-from-smoking/freedom-from-smoking-clinics.html.

[bb0010] Bader P., Boisclair D., Ferrence R. (2011). Effects of tobacco taxation and pricing on smoking behavior in high risk populations: a knowledge synthesis. Int. J. Environ. Res. Public Health.

[bb0015] Baskerville N.B., Azagba S., Norman C., McKeown K., Brown K.S. (Mar 2016). Effect of a digital social media campaign on young adult smoking cessation. Nicotine Tob. Res..

[bb0020] Biener L., Abrams D.B. (1991). The contemplation ladder: validation of a measure of readiness to consider smoking cessation. Health Psychol..

[bb0025] Brennan M. (2017). https://chinachannel.co/1017-wechat-report-users/.

[bb0030] CDC (2010).

[bb0035] CDC (2017). https://wwwcdcgov/mmwr/volumes/65/wr/mm6552a1htm?s_cid=mm6552a1_w%20.

[bb0040] Content F. (2021). https://99firms.com/blog/wechat-statistics/#gref.

[bb0045] Danaher B.G., Severson H.H., Andrews J.A. (Sep 2013). Randomized controlled trial of MyLastDip: a web-based smokeless tobacco cessation program for chewers ages 14–25. Nicotine Tob. Res..

[bb0050] Dawson G.M., Noller J.M., Skinner J.C. (Dec 2013). Models of smoking cessation brief interventions in oral health. N. S. W. Public Health Bull..

[bb0055] Dy Hu., Hong X., Li X. (2011). Oral health in China–trends and challenges. Int. J. Oral Sci..

[bb0060] Etter J.F., Duc T.V., Perneger T.V. (1999). Validity of the fagerstrom test for nicotine dependence and of the heaviness of smoking index among relatively light smokers. Addiction.

[bb0065] Fichtenberg C.M., Glantz S.A. (2002). Effect of smoke-free workplaces on smoking behaviour: systematic review. BMJ.

[bb0070] Fiorini T., Musskopf M.L., Oppermann R.V., Susin C. (2014). Is there a positive effect of smoking cessation on periodontal health?A systematic review. J. Periodontology.

[bb0075] GeneralSurgeon (2014).

[bb0080] Glanz K. (2015).

[bb0085] Hammond D., McDonald P.W., Fong G.T., Brown K.S., Cameron R. (2004). The impact of cigarette warning labels and smoke-free bylaws on smoking cessation. Can. J. Public Health.

[bb0090] Hughes J.R., Keely J.P., Niaura R.S., Ossip-Klein D.J., Richmond R.L., Swan G.E. (2003). Measures of abstinence in clinical trials: issues and recommendations. Nicotine Tob. Res..

[bb0095] Kim S.J., Marsch L.A., Brunette M.F., Dallery J. (2017). Harnessing Facebook for smoking reduction and cessation interventions: Facebook user engagement and social support predict smoking reduction. J Med Internet Res..

[bb0100] Kuo W. (2018). https://mediumcom/@williamkuo1988/china-market-research-tool-e612d559b649.

[bb0105] LATCI (2021). Let's Quit Together.

[bb0110] Luo T., Li M., Williams D. (2021). Using social media for smoking cessation interventions: a systematic review. Perspectives in Public Health.

[bb0115] Luo T., Li M.S., Williams D. (2021). Implementation of a WeChat-based smoking cessation program for chinese smokers. Int. J. Environ. Res. Public Health.

[bb0120] Luo T., Li M., Williams D. (2021). Urban and rural disparities in a WeChat-based smoking cessation intervention among chinese smokers. Int. J. Environ. Res. Public Health.

[bb0125] Namkoong K., Nah S., Van Stee S.K., Record R.A. (2018). Social media campaign effects: moderating role of social capital in an anti-smoking campaign. Health Commun..

[bb0130] Naslund J.A., Kim S.J., Aschbrenner K.A. (2017). Systematic review of social media interventions for smoking cessation. Addict. Behav..

[bb0135] Naslund J.A., Kim S.J., Aschbrenner K.A. (Oct 2017). Systematic review of social media interventions for smoking cessation. Addict. Behav..

[bb0140] Naughton F., Jamison J., Boase S. (2014). Randomized controlled trial to assess the short-term effectiveness of tailored web- and text-based facilitation of smoking cessation in primary care (iQuit in practice). Addiction.

[bb0145] Pechmann C., Pan L., Delucchi K., Lakon C.M., Prochaska J.J. (2015). Development of a twitter-based intervention for smoking cessation that encourages high-quality social media interactions via automessages. J. Med. Internet Res..

[bb0150] Pechmann C., Pan L., Delucchi K., Lakon C.M., Prochaska J.J. (2015). Development of a Twitter-based intervention for smoking cessation that encourages high-quality social media interactions via automessages. J. Med. Internet Res..

[bb0155] Pechmann C., Delucchi K., Lakon C.M., Prochaska J.J. (2017). Randomised controlled trial evaluation of Tweet2Quit: a social network quit-smoking intervention. Tobacco Control.

[bb0160] Pechmann C., Delucchi K., Lakon C.M., Prochaska J.J. (Mar 2016). Randomised controlled trial evaluation of Tweet2Quit: a social network quit-smoking intervention. Tob. Control..

[bb0165] Prochaska J.O., DiClemente C.C. (Jun 1983). Stages and processes of self-change of smoking: toward an integrative model of change. J. Consult. Clin. Psychol..

[bb0170] Prochaska J.O., Velicer W.F., DiClemente C.C., Fava J. (1988). Measuring processes of change: applications to the cessation of smoking. J. Consult. Clin. Psychol..

[bb0175] Ramo D.E., Liu H., Prochaska J.J. (2015). A mixed-methods study of young adults' receptivity to using Facebook for smoking cessation: if you build it, will they come?. Am. J. Health Promot..

[bb0180] Ramo D.E., Thrul J., Chavez K., Delucchi K.L., Prochaska J.J. (2015). Feasibility and quit rates of the tobacco status project: a Facebook smoking cessation intervention for young adults. J. Med. Internet Res..

[bb0185] Ramo D.E., Thrul J., Chavez K., Delucchi K.L., Prochaska J.J. (2015). Feasibility and Quit Rates of the Tobacco Status Project: A Facebook Smoking Cessation Intervention for Young Adults. J. Med. Internet Res..

[bb0190] Schauer G.L., Malarcher A.M., Zhang L., Engstrom M.C., Zhu S.-H. (2013). Prevalence and correlates of quitline awareness and utilization in the United States: an update from the 2009–2010 National Adult Tobacco Survey. nicotine & tobacco research.

[bb0195] Taylor G.M., Dalili M.N., Semwal M., Civljak M., Sheikh A., Car J. (2017). Internet-based interventions for smoking cessation. Cochrane Database of Systematic Reviews.

[bb0200] TCPG Treating (2008). A clinical practice guideline for treating tobacco use and dependence: 2008 update: a US public health service report. American journal of preventive medicine.

[bb0205] Tencent (2017). https://wwwtencentcom/en-us/articles/17000391523362601pdf.

[bb0210] Ussher M.H., Taylor A.H., Faulkner G.E. (2014). Exercise interventions for smoking cessation. Cochrane Database Syst. Rev..

[bb0215] WHO (2013). WHO Report on the Global Tobacco EPIDEMIC.

[bb0220] WHO (2015). http://wwwwprowhoint/china/tobacco_report_20150819_enpdf.

[bb0225] WHO (2017). http://wwwwhoint/mediacentre/factsheets/fs339/en/.

[bb0230] WHO (2018). https://extranet.who.int/ncdsmicrodata/index.php/catalog/803/download/5571.

[bb0235] Yang G., Jason H., Yang Y. (2011).

[bb0240] Yang G., Jason H., Yang Y. (2011).

[bb0245] YTD Cheung, CHH Chan, Lai C.-K.J. (2015). Using WhatsApp and Facebook online social groups for smoking relapse prevention for recent quitters: a pilot pragmatic cluster randomized controlled trial. Journal Of Medical Internet Research.

[bb0250] Zhou X., Xu X., Li J. (2018). Oral health in China: from vision to action. Int. J. Oral Sci..

