# Supplementary material for: A WeChat-based smoking cessation intervention for Chinese smokers: A pilot study
Source: Internet Interv. 2022 Feb 23;28:100511. doi: 10.1016/j.invent.2022.100511 (PMC9136339; doi:10.1016/j.invent.2022.100511)
Supplement: Supplementary file 1 — Supplementary material [file mmc1.pdf]

Supplementary 1: Recruitment Flyers Information (Advertisement)

LSU Health
NEW ORLEANS
School of Public Health

Quit Smoking Help
Q
戒烟帮助

想戒烟吗？扫码有红包！

吸烟会损害身体绝大多数器官！无数次梦想成功戒烟的您是不是想拥有更健康的身体，享受高品质的生活？

如果您：
1. 吸烟超过100支
2. 每周吸烟超过3天
3. 住在中国
4. 18岁以上
5. 每天打开微信

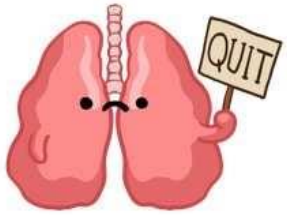

您就可以**免费**参加我们的线上活动，您只要扫码加入“我想戒烟”参与我们的研究，就可以收到2-3周视频与资讯，同时如果您完成问卷调查，还可以获得 1-20元红包。该研究是由美国路易斯安娜州立大学医学中心的研究团队来帮助您早日成功戒烟！

参与方式：请扫右侧的二维码

我想戒烟

或通过以下微信ID加入：
QuitSmokingHelp

（名额有限，报名从速）

想了解更多信息请咨询：(+86) 13764545855；(+1) 3147756241
邮箱: quitsmokinglsu@gmail.com
微信ID: QuitSmokingHelp

Recruitment Flyers Information Translated into English

Want to quit smoking? Scan the code to get lucky money!

Smoking can destroy almost every organ in the body! Would you like to have a healthier body and enjoy a better quality of life?

If you:
1. have smoked ≥100 cigarettes in your life
2. currently smoked at least 3 days per week
3. are living in China
4. are 18 years and older
5. are aactive users of WeChat (login at least once a day)

Then, you can join us for free. You only need to scan the QR code to join "I want to quit smoking" to participate into our study. You will receive smoking related videos and information for 2-3 weeks. In addition, if you complete the questionnaires, you can also receive a red packet between 1-20 RMB (0.3-3 USD) per survey. The health professional team from Louisiana State University Health Sciences Center will help you to find a way to quit smoking. Cheer up! You can do this!

How to participate: please scan the QR code
Or join by WeChat ID: QuitSmokingHelp

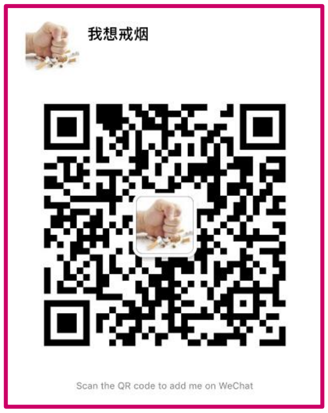

Limited registration

For more information, please contact: (+86) 13764545855；(+1) 3147756241
Email: quitsmokinghelp@gmail.com
WeChat ID: QuitSmokingHelp

Supplementary 2: Conceptual Model

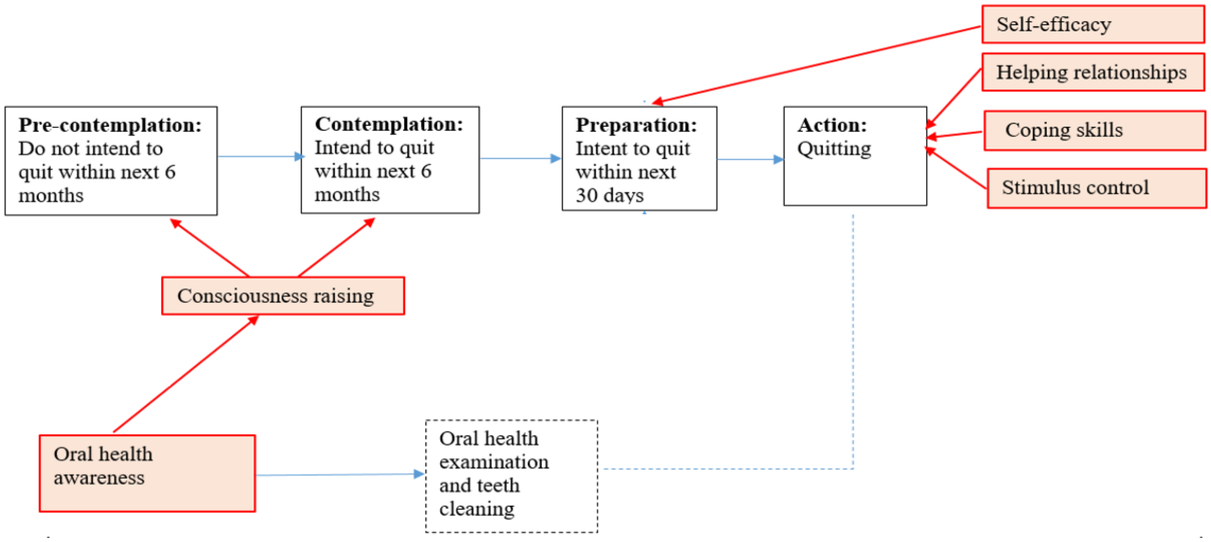

Pre-contemplation, contemplation, preparation, and action are the 4 stages of Stage of Change, which is part of the Transtheoretical Model (TTM).
Highlight boxes are the six intervention content categories, which adapted from the TTM. Our intervention messages were developed based on these intervention content categories.

Supplementary 3: Intervention Information Delivery Schedule

Time posted	Topic	Delivered group
	**Intervention Wk1**	
**Wk1day1 (Aug 5)**	Harms of smoking & Benefits of quitting smoking	Group 1/2
**Wk1day2 (Aug 6)**	Reasons to quit & What is stopping you from quitting smoking?	Group 1/2
**Wk1day3 (Aug 7)**	Understanding of nicotine & smoking cessation medications	Group 1/2
**Wk1day4 (Aug 8)**	Success stories from Jackie Chan & Success stories from Peiwei Ni	Group 1/2
**Wk1day5 (Aug 9)**	How to make a quit plan & self-management approaches	Group 1/2
**WK1day5-day7 (Aug 9 – Aug 11)**	Process evaluation 1	Group 1/2
	**Intervention Wk2**	
**Wk2day1 (Aug 12)**	Setting up the social support you need & Seeking social support from us	Group 1/2
**Wk2day2 (Aug 13)**	Identifying common triggers and techniques & Saying no to people who hand you cigarettes	Group 1/2
**Wk2day3 (Aug 14)**	Coping with withdrawal symptoms & Preventing slips and relapse	Group 1/2
**Wk2day4 (Aug 15)**	Physical relaxation techniques & Imaginary relaxation techniques	Group 1/2
**Wk2day5 (Aug 16)**	Weight management in diet & in exercise	Group 1/2
**WK2day5-day7 (Aug 16 – Aug 18)**	Process evaluation 2 + immediate outcome	Group 1/2
	**Intervention Wk 3**	
**Wk3day1 (Aug 19)**	Smoking and oral health & Oral health problems	Group 2
**Wk3day2 (Aug 20)**	How to take care of teeth & Benefits of receiving routine teeth cleanings and dental checks	Group 2
**Wk3day3 (Aug 21)**	The necessity of ultrasonic teeth cleaning & common questions about teeth cleaning	Group 2
**WK3day3-day5 (Aug 21 – Aug 23)**	Process evaluation 3	Group 2
**Sept 6-Sept 8**	4-weeks follow-up assessment	Group 1/2/3
	**Intervention for Control Group Wk1**	
**Wk1day1 (Sept 9)**	Harms of smoking & Benefits of quitting smoking	Group 3
**Wk1day2 (Sept 10)**	Reasons to quit & What is stopping you from quitting smoking?	Group 3
**Wk1day3 (Sept 11)**	Understanding of nicotine & smoking cessation medications	Group 3
**Wk1day4 (Sept 12)**	Success stories from Jackie Chan & Success stories from Peiwei Ni	Group 3
**Wk1day5 (Sept 13)**	How to make a quit plan & self-management approaches	Group 3
	**Intervention for Control Group Wk2**	
**Wk2day1 (Sept 16)**	Setting up the social support you need & Seeking social support from us	Group 3
**Wk2day2 (Sept 17)**	Identifying common triggers and techniques & Saying no to people who hand you cigarettes	Group 3
**Wk2day3 (Sept 18)**	Coping with withdrawal symptoms & Preventing slips and relapse	Group 3
**Wk2day4 (Sept 19)**	Physical relaxation techniques & Imaginary relaxation techniques	Group 3
**Wk2day5 (Sept 20)**	Weight management in diet & in exercise	Group 3

Group 1: Standard Group, Group 2: Enhanced Group, Group 3: Waitlist Group.

Two examples of the intervention messages are shown below:

鉴别常见的诱因以及应该技巧

Identifying Common Trigger and Coping Techniques

如果你：

被吸烟的人包围了

IF YOU ARE

Being around other people who smoke.

你可以：

去一个不允许吸烟的地方，或者告诉你的朋友你正在戒烟

YOU CAN

Go to a place where smoking is not allowed, OR tell friends who smoke that you are trying to quit.

如果你：

觉得无聊想抽烟

IF YOU ARE

Feeling bored

你可以：

找新事情做，比如阅读、散步、或者培养一种新的爱好

YOU CAN

Find new ways to occupy your time.
For example: read, take walks, or strat a hobby.

如果你：

喝酒的时候想抽烟

IF YOU ARE

Drinking alcohol

你可以：

在你试图戒烟的那段时间里避免喝酒和聚会

YOU CAN

Avoid drinking alcoholic beverage while you are trying to quit. Avoid drinking parties.

如果你：

饿的时候想抽烟

IF YOU ARE

Feeling hungry

你可以：

吃健康的零食，
或者喝水，
做运动也有帮助。

YOU CAN

Have a healthy snack or drink some water.
Exercise can also help.

如果你：

喝咖啡的时候想抽烟

IF YOU ARE

Drinking coffee

你可以：

把喝咖啡换成喝茶，或者用通常夹烟的那只手握咖啡杯。

YOU CAN

Switch to tea, or hold your cup with the hand you normally use to hold a cigarette.

如果你：

打电话的时候想抽烟

IF YOU ARE

Taking on the phone.

你可以：

在通常夹烟手里放点其他的东西，比如笔，也可以在废纸上涂鸦。

YOU CAN

Put something else in your hand, such as a pen.
Doodle on scrap paper.

如果你：

看电视的时候想抽烟

IF YOU ARE

Watching TV

你可以：

避免坐经常坐的椅子或沙发，同时在手边放一点健康的零食。

YOU CAN

Avoid sitting in your usual chair. Keep healthy snacks on hand.

如果你：

吃过饭以后想抽烟

IF YOU ARE

Finishing a meal

你可以：

立马离开餐桌。马上刷牙。饭后散步或快走。

YOU CAN

Leave the table immediately.
Brush your teeth right after eating.
Take a walk.

如果你：

早晨起床后想抽烟

IF YOU ARE

Waking up in the morning.

你可以：

醒来以后立马去刷牙、洗澡、吃早饭。运动也会有帮助。

YOU CAN

Take a shower, eat breakfast or brush your teeth as soon as wake up. Exercise can help.

如果你：

开车上下班
路上想抽烟

IF YOU ARE

Driving to and from work

你可以：

放你喜欢的音乐并跟着哼。
选另一条不熟悉的路上下班。
搭乘不吸烟的人。
把你的烟放到后备箱里。
也可以考虑用公共交通。

YOU CAN

Play your favorite music or sing along with the radio.
Take a different rout.
Carpool with a nonsmoker.
Put cigarettes on the trunk.
Take public transportation, if possible.

我想戒烟

Scan the QR code to add me on WeChat

在戒烟的路上，
你有什么问题，
可以随时与我们联系，
我们一直都在。

Feel free to contact with us and we will always there and support you for quitting smoking!

谢谢观看

Thank you for watching!

视频与PPT制作：素无尘
Slides and video design: Suwuchen

内容来源：路易斯安那烟草控制倡议
Text source: Louisiana Tobacco Control Initiative

Quit Smoking Help
戒烟帮助

如何应对戒断症状？

How To Cope With Withdrawal Symptoms

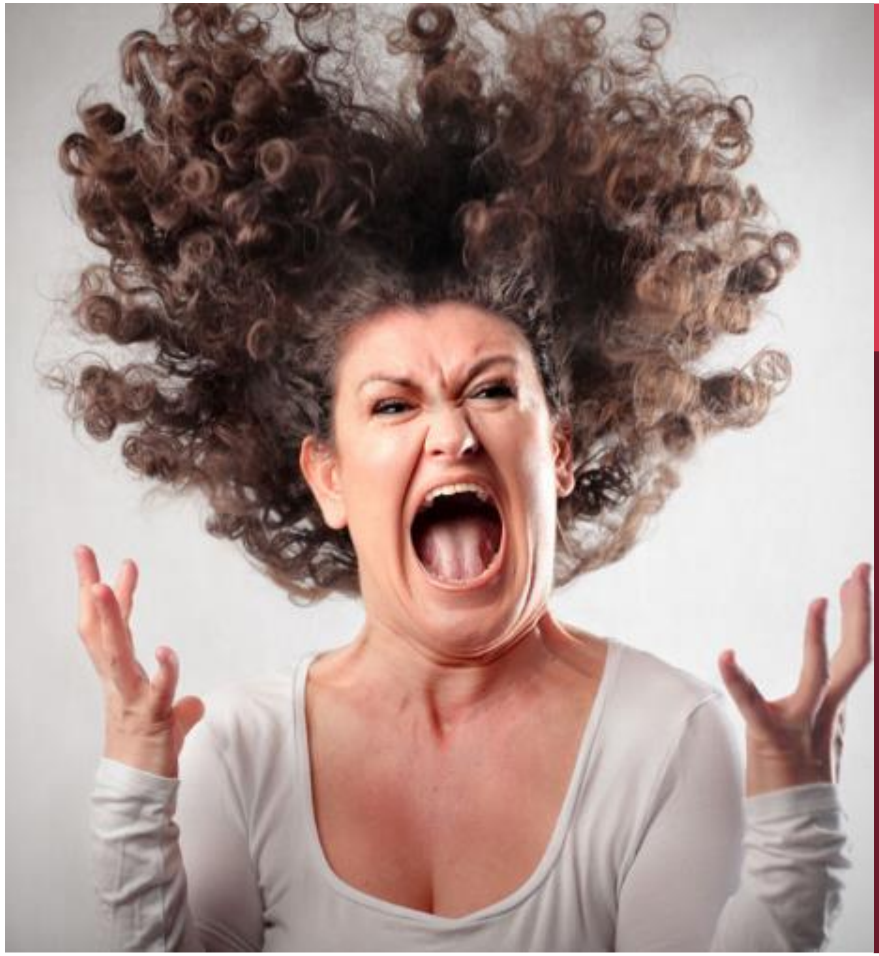

感觉烦躁、喜怒无常或紧张

Feeling irritable, moody, or tense

散步、骑车、游泳等做有利于放松的运动，或者打电话给支持你戒烟的人。

Walking, biking, swimming.
Or call a person who support you quitting smoking.

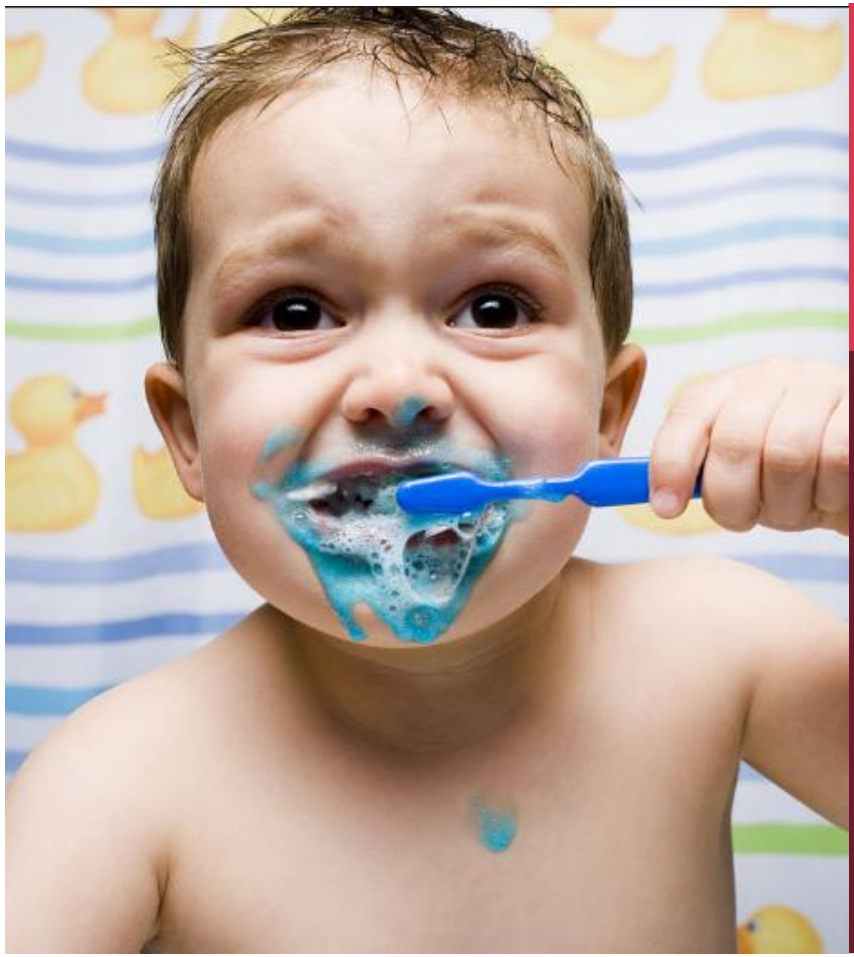

对抽烟的渴望

Craving

刷牙、喝水、散散步

Brush your teeth, drink a glass of water, take a walk or exercise.

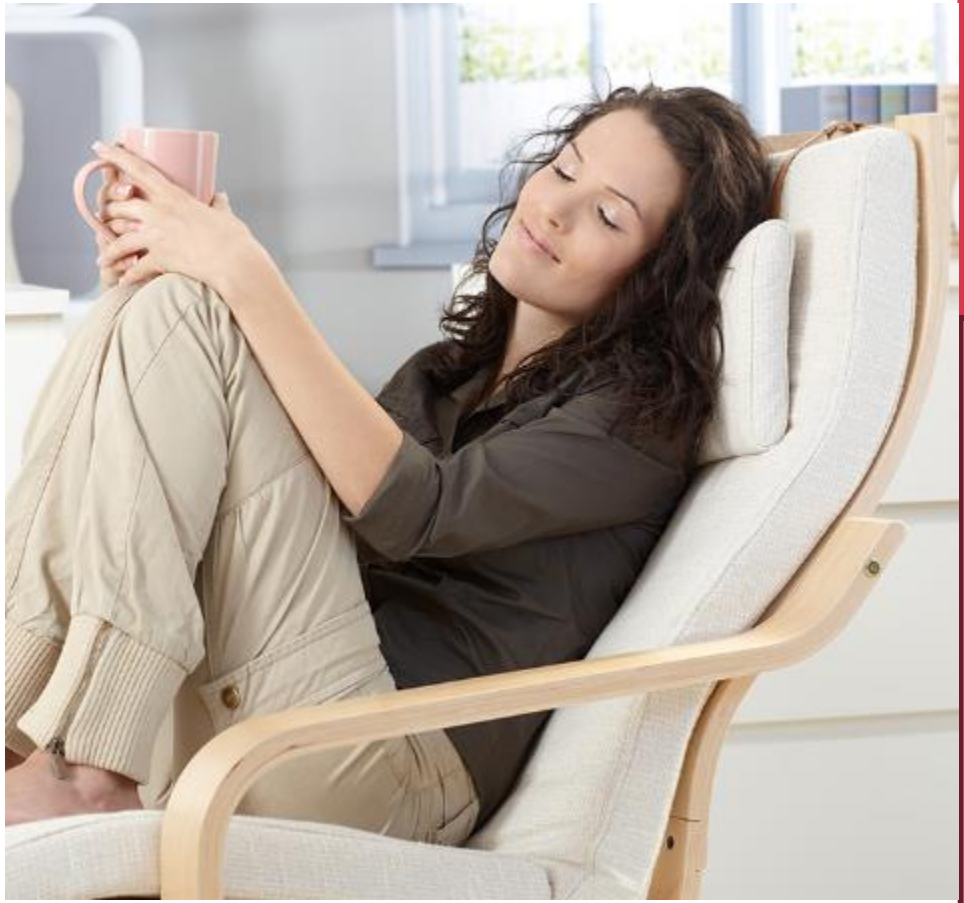

头晕、眼花

Headaches or dizziness

坐下或躺下，闭上眼睛，并告诉自己，你可以。

Sit or lie down and close your eyes, and tell yourself you can do this.

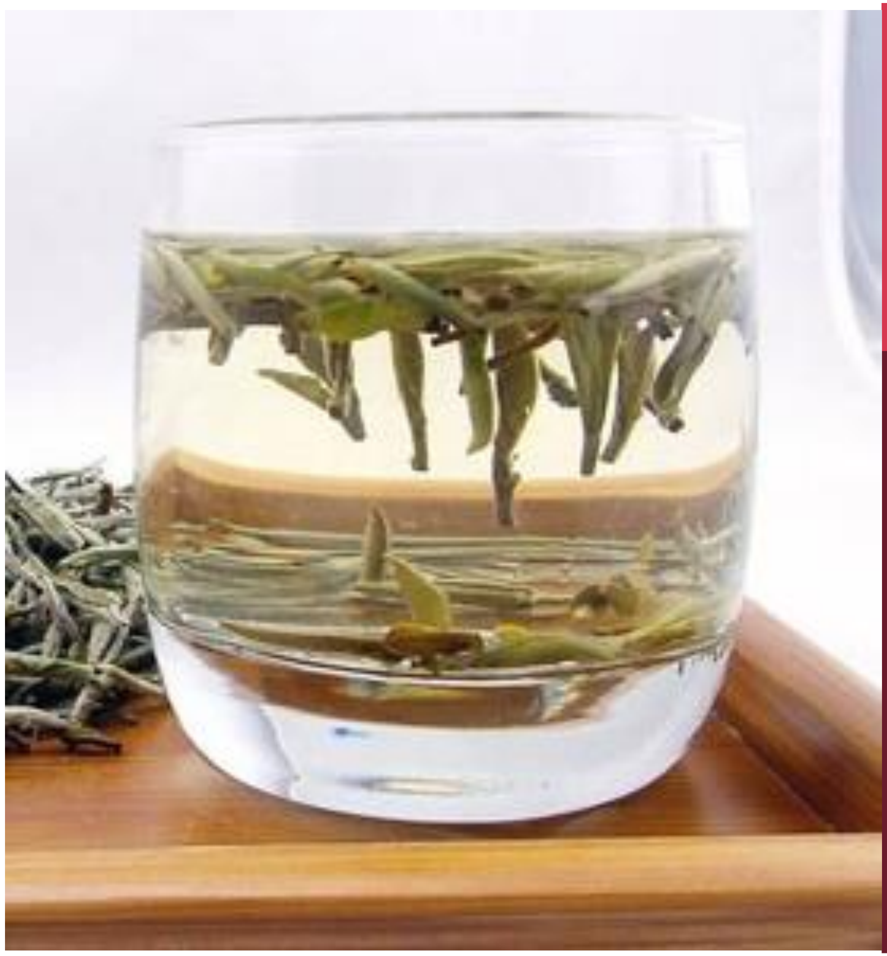

嘴巴干、喉咙痛

Dry mouth or sore throat

喝水或者喝绿茶加蜂蜜，并且把茶叶嚼碎吞下。

Drink a glass of water or drink green tea with honey, chew and swallow the leaves.

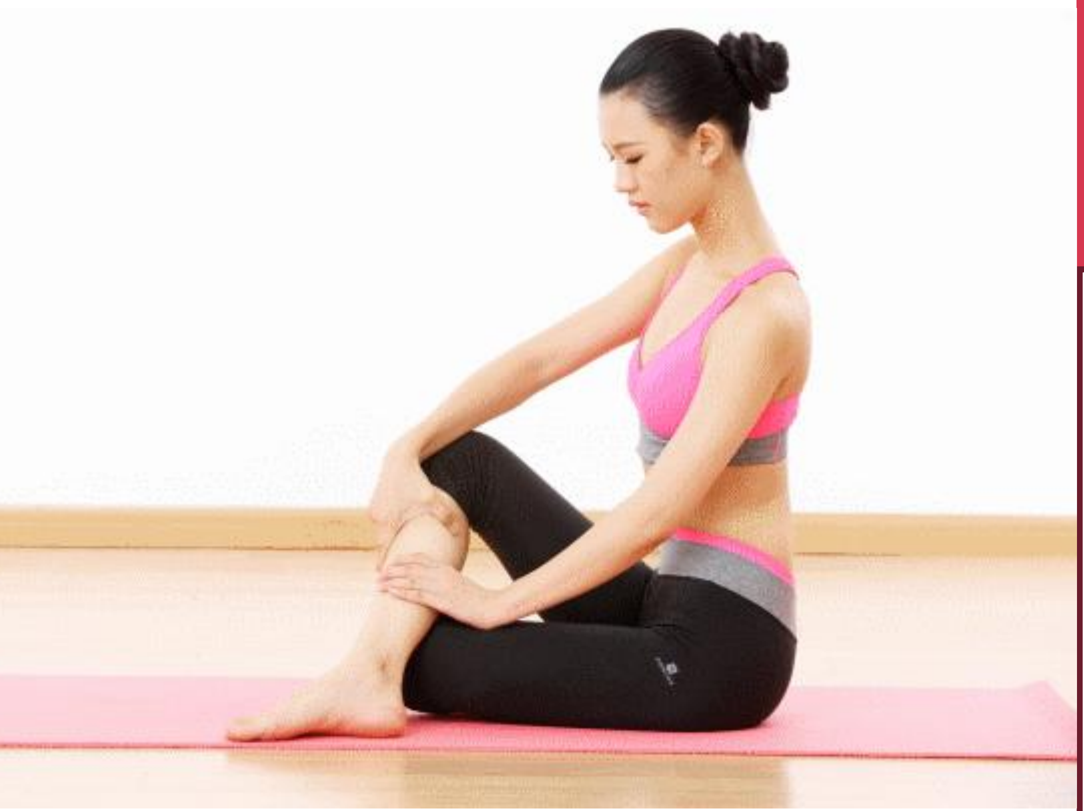

失眠

Insomnia

从下午开始避免含有咖啡因的食物，比如茶、巧克力。睡前可以做一些轻量运动。

Avoid caffeine in the afternoon and evening, and do some physical activities.

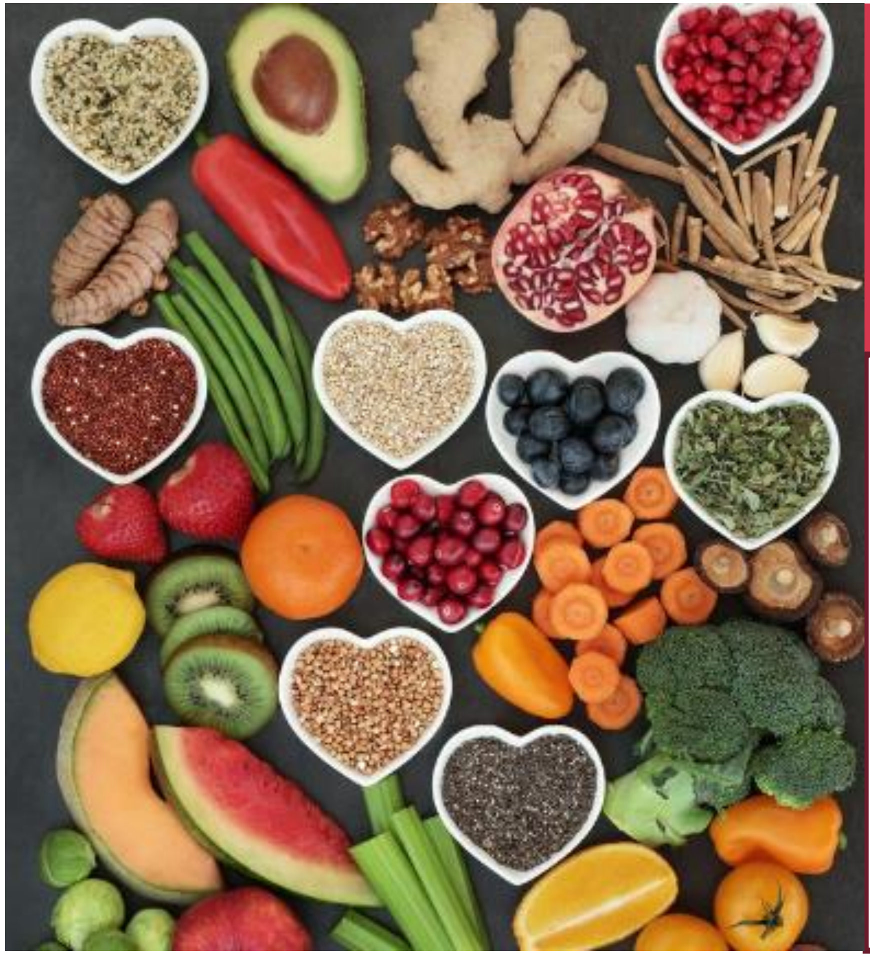

肚子痛、便秘

Upset stomach and/or constipation

每天喝足量的水（6-8杯），
多吃水果、蔬菜和全谷物食物。

Try to drink 6-8 glasses of water a day, eat more fruits, vegetables, and whole-grain foods.

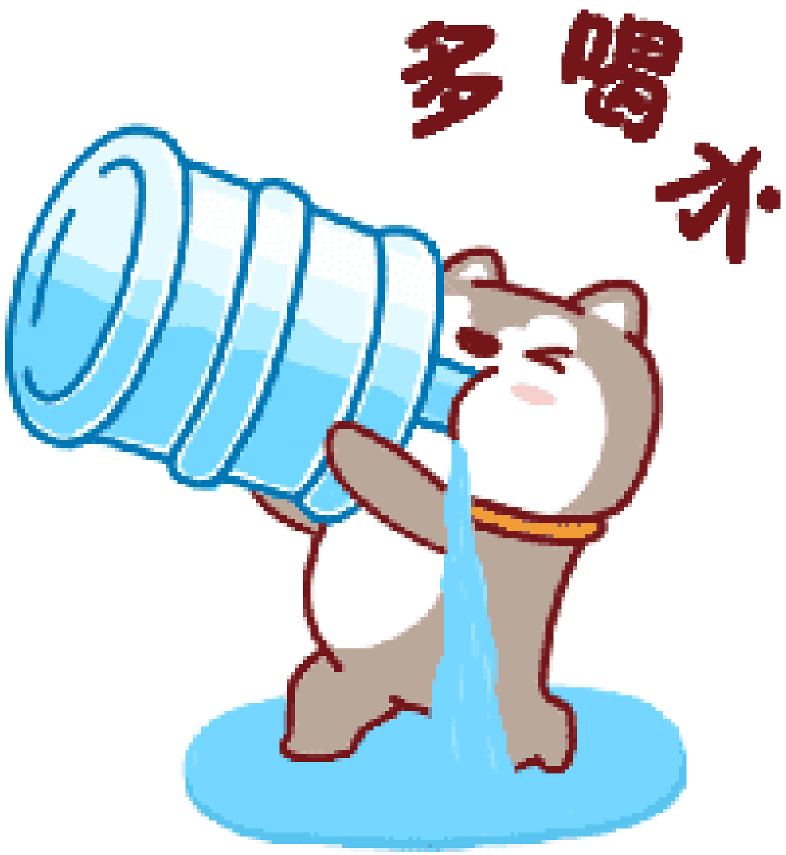

咳嗽

Coughing

喝水、果汁、热茶

Drink water, juice, warm herbal tea.

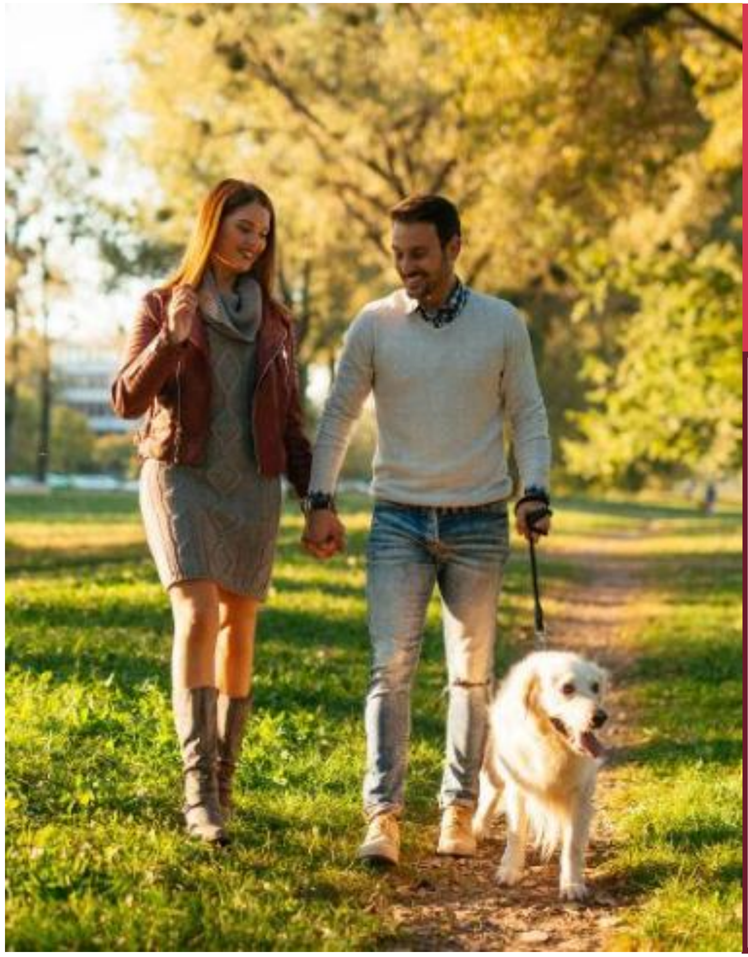

嗜睡、疲劳

Drowsiness or fatigue

外出散步获取一些新鲜空气，或者做一些放松的活动，也可以小睡一会。

Take a walk and get some fresh air, do something relaxing, or take a nap.

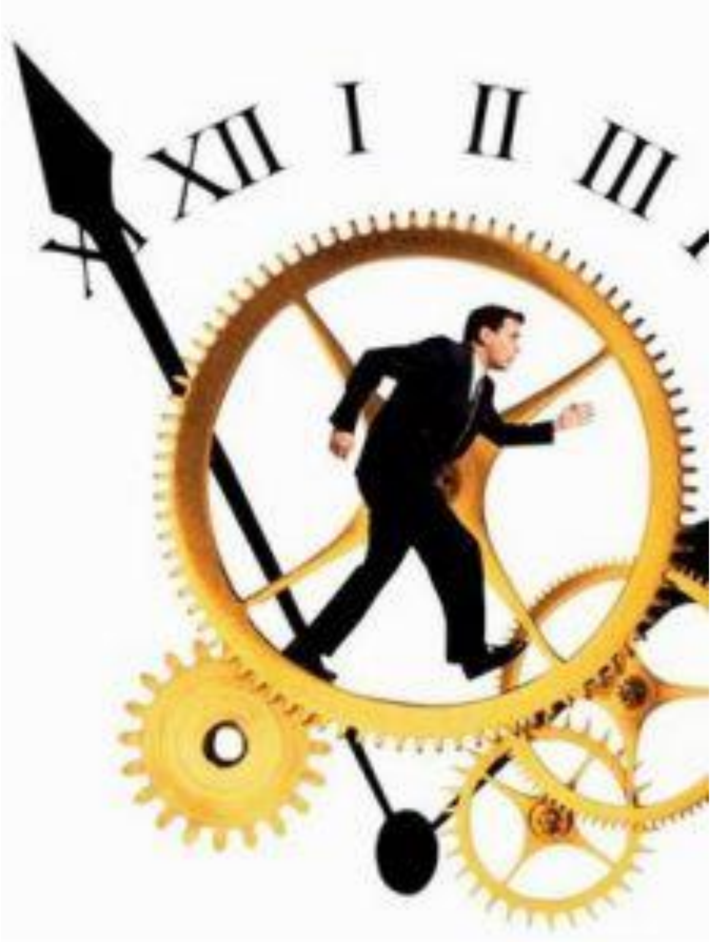

注意力难以集中

Difficulty concentrating

减压
管理好时间
适当休息

Reduce stress, manage your time, take breaks.

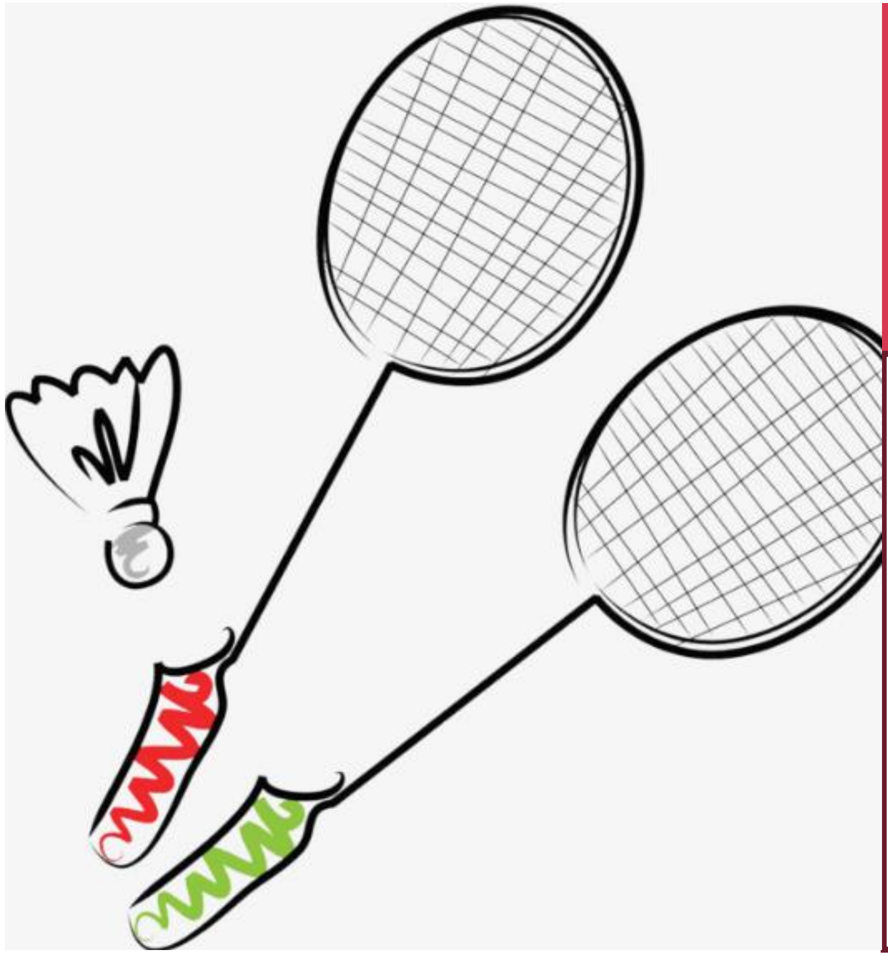

胃口大好，怕会长胖

Increased appetite, afraid of weight gain

多喝水
吃卡路里含量低的食物
多做运动

Drink water, eat low-calorie snacks, and do exercise.

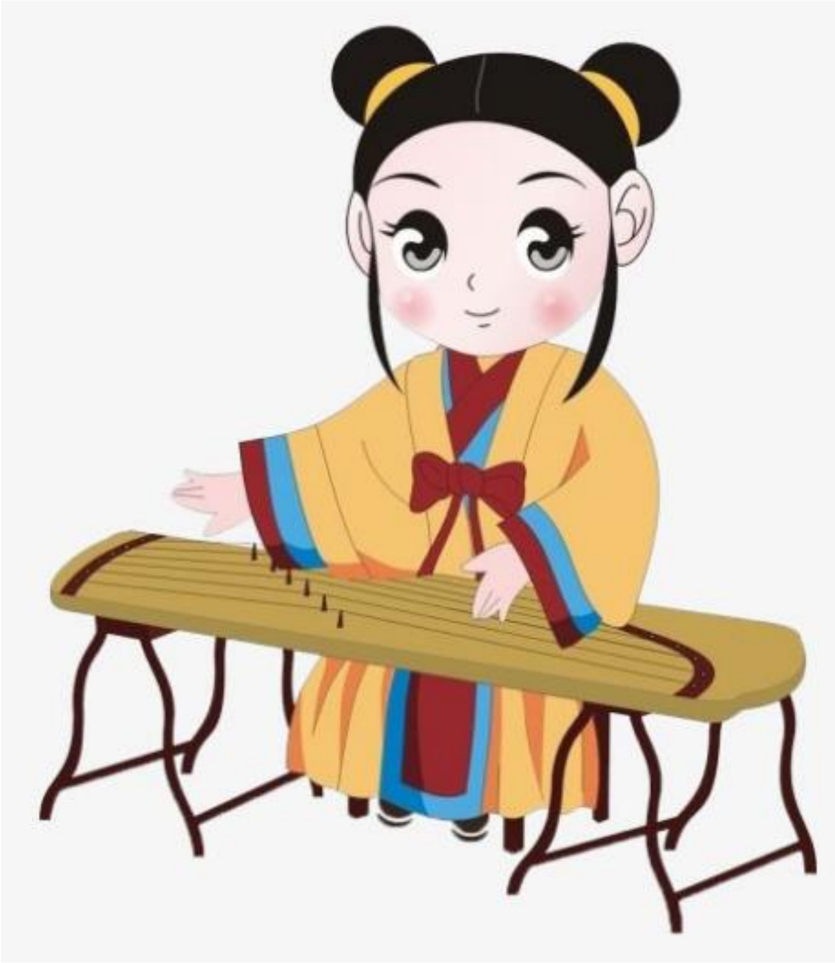

坐立不安

Feeling restless

外出散步
适当运动
开始一个新的兴趣爱好

Go for a walk, exercise, or do a hobby.

我想戒烟

Scan the QR code to add me on WeChat

在戒烟的路上，
你有什么问题，
可以随时与我们联系，
我们一直都在。

Feel free to contact us and we will always support your efforts to quit smoking!

非常感谢您的收看

Thank you for watching!

视频与PPT制作：素无尘
Slides and video design: Suwuchen

内容来源：路易斯安那烟草控制倡议
Text source: Louisiana Tobacco Control Initiative

图片来源：谷歌
All pictures from Google images

Quit Smoking Help 戒烟帮助

Supplementary 4: Content Measurement

Content measurements were evaluated pre-and post-intervention. We measured all 6 content categories described as follows (See table S1).

1) Consciousness Raising

Dr. Prochaska and his colleagues developed a measurement for process of change in the field of smoking cessation [1]. They used a 4-item questionnaire to measure consciousness raising. We adapted two items in this questionnaire to fit our study; with the meaning of each item remaining the same. The old and updated versions are as follows:

a) I recall **articles** dealing with the problem of quitting smoking. (Yes/No) → I recall **information** dealing with the problem of quitting smoking. (Yes/No)
b) I think about information **from articles** and advertisements on how to quit smoking. (Yes/No) → I think about information **from WeChat** on how to quit smoking. (Yes/No)
c) I recall information people have given me on how to quit smoking. (Yes/No) → Kept the same
d) I recall information people have personally given me on the benefits of quitting smoking. (Yes/No) → Kept the same

2) Self-Efficacy

Self-efficacy has been applied to smoking cessation for several decades, and many studies have demonstrated that it works well in regards to smoking cessation [2-4]. There are several common self-efficacy measurements: a 31 item [5], 12 item [6], 9 item [7, 8], and 1 item questionnaire [3, 9, 10]. However, Dr. Gwaltney and his colleagues have shown that the relationship between the number of items included in the self-efficacy assessment and the magnitude of the effective size is not significant[4]. The 1-item measurement has its own strengths, for instance, being easy to operate and not labor-intensive. Thus, many studies have adopted the 1-item question to measure self-efficacy, such as the studies

conducted by Dr. Abrams et al., [3] by Dr. Lipkus et al., [10] and by Dr. Gritz et al., [9]. In addition, these studies have numerous citations (ranging between 60-150), indicating that the study results have been accepted by the academic community. Therefore, we also used the single item questionnaire to measure self-efficacy: 'How confident are you that you will be able to stop smoking in the next 6 months?' Responses ranged from 1 = 'Extremely confident' to 5 = 'Not confident'.

3) Helping Relationships

A study conducted by Dr. Prochaska and his colleagues, used 4-item questionnaire to measure helping relationships [1]. We adopted this questionnaire into our study as well, as follows:

a) Special people in my life accept me the same whether I smoke or not. [1] (Yes/No)
b) I can be open with at least one special person about my experience with smoking. [1] (Yes/No)
c) I have someone who is willing to listen to me when I need to talk about my smoking. [1] (Yes/No)
d) I have someone who I can count on when I am having problems with smoking. [1] (Yes/No)

4) Stimulus Control

Dr. Prochaska and his colleagues (1988) also used a 4-item questionnaire to measure stimulus control. [1] We adopted this questionnaire into our study as well, as follows:

a) I remove things from my home that remind me of smoking. [1] (Yes/No)
b) I put things around my home that remind me not to smoke. [1] (Yes/No)
c) I remove things from my place of work that remind me of smoking. [1] (Yes/No)
d) I keep things around my place of work that remind me not to smoke. [1] (Yes/No)

5) Coping Skills

Our measurement of coping skills was based on the Brief COPE inventory, which has been widely accepted, with more than 3000 citations. [11] Each coping strategy was assessed using two items, rated on

a four-point Likert-type scale, ranging from 1 = 'I have not been doing this at all' to 4 = 'I have been doing this a lot.' [11] We adjusted the "active coping" measurement into our study, as follow:[11, 12]

a) I have been concentrating my efforts on doing something about the situation I am in. [11]→I have been concentrating my efforts on doing something about quitting smoking.
b) I have been taking action to try to make the situation better. [11] →I have been taking action to try to make my situation better by quitting smoking.

6) Oral Health Awareness

An 8-item questionnaire that has been used to measure the oral health behavior attitude of Chinese adults was used to measure dental knowledge. [13] The questionnaire was as follows:

a) Brushing one's teeth prevents tooth decay. (Agree, Disagree, and Don't know)
b) Brushing one's teeth makes for healthy gums. (Agree, Disagree, and Don't know)
c) Using floss to clean the spaces between one's teeth is no guarantee for healthy gums. (Agree, Disagree, and Don't know)
d) Tobacco is bad for the teeth and mouth. (Agree, Disagree, and Don't know)
e) Sweet products are bad for the teeth. (Agree, Disagree, and Don't know)
f) Fluoridated drinking water protects your teeth. (Agree, Disagree, and Don't know)
g) Using fluoride is a harmless way of preventing tooth decay. (Agree, Disagree, and Don't know)
h) Going to the dentists will solve problems I have with my teeth, gums or dentures. (Agree, Disagree, and Don't know)

Because we need to adapt and transform this questionnaire to measure dental knowledge in our study, we brought this questionnaire to group of experts for discussion. Since the original responses of "Agree", "Disagree", and "Don't know" for each question measured attitudes in the cited study, [13] we will change the answers to "Yes", "No" and "Don't know" for our measurement of dental knowledge.

For item C, “Using floss to clean the spaces between one’s teeth is no guarantee for healthy gums”, we will change the language, but keep the meaning of the sentence the same. The updated language for item C will be “"Flossing regularly is one way to maintain healthy gums".

According to Petersen et al., items A, B, C, and E have very high rates of “Agree” responses, at roughly 90%. Items A and B are similar, as well as items D and E. Thus, we kept one question from items A and B, and kept one question from items D and E. Therefore, we decided on the flowing 6-item questionnaire that was used in our study:

1) Brushing your teeth makes for healthy gums. (Yes, No, and Don’t know)
2) Flossing regularly is one way to maintain healthy gums. (Yes, No, and Don’t know)
3) Tobacco is bad for your teeth and mouth. (Yes, No, and Don’t know)
4) Fluoridated drinking water protects your teeth. (Yes, No, and Don’t know)
5) Using fluoride is a harmless way of preventing tooth decay. (Yes, No, and Don’t know)
6) Going to the dentist will solve the problems you have with your teeth, gums or dentures. (Yes, No, and Don’t know)

Participants who respond “Yes” will receive 1 point and participants who respond “No” or “Don’t know” will receive 0 points.

Oral health awareness may influence smoking cessation outcomes through the mediator of consciousness raising; however, increasing oral health awareness may also have direct impacts on the intention to quit. Thus, we included one question to measure whether oral health is a cue for smokers to quit. Cues to action are an important concept from the Health Belief Model (HBM), and can be either internal or external, with mass media communication constituting an external form. [14] These cues to action can appear via WeChat messages, televised news, or public service announcements.[14]

According to Jones et al., although the Cues to Health Action Questionnaire contains 32 items, each cue has its own question (each question represents a cue that could prompt an individual to take

health-promoting actions) [15]. Thus, we used the following item to measure whether oral health information is a significant cue for smoking cessation action.

a) Hearing through WeChat about how smoking is related to oral health makes me think about quitting smoking. [15]

   Responses was evaluated on a four-point scale with 0 = not at all, 1 = possibly likely, 2 = moderately likely, and 3 = very likely.

The entire content measurement section is summarized in Table S1.

Table S1: Content Measurements and Cut-off Criteria

**Content**	**Measurement**
**Consciousness raising**	1) I recall information dealing with the problem of quitting smoking. [1] (Yes/No)<br>2) I think about information on how to quit smoking. [1] (Yes/No)<br>3) I recall information from WeChat people have given me on how to quit smoking. [1] (Yes/No)<br>4) I recall information people have personally given me on the benefits of quitting smoking. [1] (Yes/No)
**Self-efficacy**	1) How confident are you that you will be able to stop smoking in the next 6 months? [3, 9, 10] The reliability coefficient was demonstrated to be 0.92.[16]<br>Responses are anchored with 1 = 'Extremely confident' to 5 = 'Not confident'.
**Helping relationships**	1) Special people in my life accept me the same whether I smoke or not. [1] (Yes/No)<br>2) I can be open with at least one special person about my experience with smoking. [1] (Yes/No)<br>3) I have someone who is willing to listen to me when I need to talk about my smoking. [1] (Yes/No)<br>4) I have someone who I can count on when I am having problems with smoking. [1] (Yes/No)
**Stimulus control**	1) I remove things from my home that remind me of smoking. [1] (Yes/No)<br>2) I put things around my home that remind me not to smoke. [1] (Yes/No)<br>3) I remove things from my place of work that remind me of smoking. [1] (Yes/No)<br>4) I keep things around my place of work that remind me not to smoke. [1] (Yes/No)
**Coping skills**	1) I have been concentrating my efforts on doing something about quitting smoking. [11]<br>2) I have been taking action to try to make my situation better by quitting smoking. [11]
**Oral health awareness**	1) Brushing your teeth makes for healthy gums. [13]<br>2) Flossing regularly is one way to maintain healthy gums. [13]<br>3) Tobacco is bad for your teeth and mouth. [13]<br>4) Fluoridated drinking water protects your teeth. [13]<br>5) Using fluoride is a harmless way of preventing tooth decay. [13]<br>6) Going to the dentist will solve the problems you have with your teeth, gums, or dentures. [13]<br><br>Extra cues to action<br>1) Hearing through WeChat about how smoking is related to oral health makes me think about quitting smoking. [15]

	Responses will be evaluated on a four-point scale with 0 = not at all, 1 = possibly likely, 2 = moderately likely, and 3 = very likely.

References:

1. Prochaska, J.O., et al., *Measuring processes of change: applications to the cessation of smoking.* Journal of consulting and clinical psychology, 1988. **56**(4): p. 520.
2. Prochaska, J.O. and C.C. DiClemente, *Stages and processes of self-change of smoking: toward an integrative model of change.* J Consult Clin Psychol, 1983. **51**(3): p. 390-5.
3. Abrams, D.B., et al., *Stages of change versus addiction: a replication and extension.* Nicotine & Tobacco Research, 2000. **2**(3): p. 223-229.
4. Gwaltney, C.J., et al., *Self-efficacy and smoking cessation: a meta-analysis.* Psychology of Addictive Behaviors, 2009. **23**(1): p. 56.
5. Velicer, W.F., et al., *Relapse situations and self-efficacy: An integrative model.* Addictive behaviors, 1990. **15**(3): p. 271-283.
6. Etter, J.F., et al., *Development and validation of a scale measuring self-efficacy of current and former smokers.* Addiction, 2000. **95**(6): p. 901-913.
7. Dijkstra, A. and G. Ten Wolde, *Ongoing interpretations of accomplishments in smoking cessation: positive and negative self-efficacy interpretations.* Addictive behaviors, 2005. **30**(2): p. 219-234.
8. Li, S., et al., *Mediation of smoking abstinence self-efficacy on the association of nicotine dependence with smoking cessation.* The European Journal of Public Health, 2014. **25**(2): p. 200-204.
9. Gritz, E.R., et al., *A randomized trial of a self-help smoking cessation intervention in a nonvolunteer female population: testing the limits of the public health model.* Health Psychology, 1992. **11**(5): p. 280.
10. Lipkus, I.M., P.R. Lyna, and B.K. Rimer, *Using tailored interventions to enhance smoking cessation among African-Americans at a community health center.* Nicotine & Tobacco Research, 1999. **1**(1): p. 77-85.
11. Carver, C.S., *You want to measure coping but your protocol'too long: Consider the brief cope.* International journal of behavioral medicine, 1997. **4**(1): p. 92.
12. McGee, R., et al., *Tobacco smoking in adolescence predicts maladaptive coping styles in adulthood.* nicotine & tobacco research, 2013. **15**(12): p. 1971-1977.
13. Petersen, P.E., B. Peng, and B.J. Tai, *Oral health status and oral health behaviour of middle-aged and elderly people in PR China.* International Dental Journal, 1997. **47**(6): p. 305-312.
14. Glanz, K., *Health behavior: Theory, research, and practice*. 2015: John Wiley & Sons.
15. Jones, T., M.C. Fowler, and D. Hubbard, *Refining a tool to measure cues to action in encouraging health-promoting behavior—the CHAQ.* American Journal of Health Promotion, 2000. **14**(3): p. 170-173.
16. Ham, O.K., *Stages and processes of smoking cessation among adolescents.* Western Journal of Nursing Research, 2007. **29**(3): p. 301-315.

Supplementary 5: Demographic Information and Smoking Behaviors at Baseline Comparison between Completion and Attrition

Variables	Follow-up Completion (n=216)		Follow-up Attrition (n=187)		p-value
	n	%	n	%	
**Age Category**					.13
18-24	73	34.2	48	26.8	
25-29	58	27.2	42	23.5	
30-39	54	25.4	54	30.2	
≥40	28	13.2	35	19.6	
**Sex**					.01
Male	200	92.6	158	84.5	
Female	16	7.4	29	15.5	
**Household Income Annually[a] in ¥**					.52
<50,000	71	32.9	74	39.6	
50,000-99,999	67	31.0	49	26.2	
100,000-199,999	53	24.5	42	22.5	
≥200,000	25	11.6	22	11.8	
**Self-Reported Living Area[b]**					.98
Urban	124	57.4	109	58.3	
Suburban	58	26.9	49	26.2	
Rural	34	15.7	29	15.5	
**Education Level**					.24
High School or Less	77	35.7	76	40.6	
Associated College	77	35.7	52	27.8	
College and Above	62	28.7	59	31.6	
**Marital Status**					.58
Married	127	58.8	115	61.5	
Single[c]	89	41.2	72	38.5	
**Occupation**					.96
Business[d]	100	46.3	81	43.3	
Government/agency officers/ professional staff [e]	40	18.5	35	18.7	
Labor Workers [f]	28	13.0	26	13.9	
Self-Employed and Other [g]	48	22.2	45	24.1	
**BMI[h]**					.21
Under-weight and Normal Weight	130	60.5	101	54.3	
Overweight and Obese	85	39.5	85	45.7	
**Age of Smoking Initiation (Mean, SD)**	18.1	3.9	18.1	4.2	.93
**Stage of change**					.56
Pre-Contemplation	15	6.9	9	4.8	
Contemplation	101	46.8	84	44.9	
Preparation	100	46.3	94	50.3	
**Smoked in the past 24 hours**					.74
Yes	204	94.4	178	95.2	
No	12	5.6	9	4.8	
**Smoked in the past 7 days**					
Yes	216	100	187	100	
No	0	0	0	0	
**Daily Cigarettes Use**					.09
10 or Less	95	44.0	67	35.8	
11-20	96	44.4	83	44.4	
21-30	19	8.8	25	13.4	
31 or More	6	2.8	12	6.4	
**Nicotine Dependence (Mean, SD)**	5.1	2.4	5.4	2.5	.35

[a] The current exchange rate for USD to RMB is as follows: ￥6.5=$1; ￥<20,000=<$3,077; ￥ 20,000-49,999 = $3,077-$7,692; ￥ 50,000-99,999 = ($7,692-$15,384); ￥100,000-199,999 = $15,384-$30,769; ￥ >200,000 = >$30,769.

[b] "Urban" includes people who are living in prefecture-level cities or county-level cities; "Suburban" includes people who are living in the areas beyond a city's border; "Exurban" includes people who are living in towns; and "Rural" includes people who are living in villages.

[c] "Single" includes never married, widowed, divorced, and living with partner.

[d] "Business" includes managers, general office staff, and business service workers (e.g., salesmen, shop clerks, waiters, etc).

[e] "Professional Staff" includes doctors, teachers, lawyers, journalists, etc.

[f] "Labor Workers" includes factory workers, and farmers/foresters/fishermen.

[g] "Self-Employed and Other" includes self-employed, freelancers, retired, unemployed, students, and others.

[h] Asian BMI standards are as follows: Underweight and Normal Weight (BMI≤22.9), Overweight and Obese (BMI≥23).
